# Supplementary material for: The effects of weak selection on neutral diversity at linked sites
Source: Genetics. 2022 Feb 12;221(1):iyac027. doi: 10.1093/genetics/iyac027 (PMC9071562; doi:10.1093/genetics/iyac027)
Supplement: iyac027_Supplementary_Data [file iyac027_supplementary_data.zip › Supplemental_Figure_Captions_GENETICS-2022-305040.pdf]

Figure S1. The mean sum of the diversity per generation over the course of the fixation of a new mutation (H^*) as a function of the absolute value of the scaled selection coefficient, assuming autosomal inheritance and a Wright-Fisher model of genetic drift with population size N. The cases with negative selection have a dominance coefficient h = 0.1 and those with positive selection have h = 0.9. The results for two different values of N are shown. The black curves are the values from the approximation of Equation (4), and the blue curves are the values from numerical integration of the diffusion equation results (Equation A6). The horizontal lines are the neutral values from the numerical integrations.

Figure S2. The mean sum of the diversity per generation over the course of the loss of a new mutation (H^{**}) as a function of the absolute value of the scaled selection coefficient, assuming autosomal inheritance and a Wright-Fisher model of genetic drift with population size N. The cases with negative selection have a dominance coefficient h = 0.1 and those with positive selection have h = 0.9. The results for two different values of the population size, N, are shown. The black curves are the values from the approximation of Equation (4), and the blue curves are the values from numerical integration of the diffusion equation results (Equation A7). The horizontal lines are the neutral values from the numerical integrations.

Figure S3. Means of the sums of the diversity per generation over the course of fixation plotted against the mean time to fixation (upper panel), and over the course of the loss of a new mutation plotted against the mean time to loss (lower panel). The data for N = 50 from the integration result is Figures S1 and S2 were used for these plots. The results in the upper panel are identical for $\gamma \geq$ and h = 0.9, and for $\gamma \leq 0$ and h = 0.1. The plot for losses with $\gamma \leq 0$ and h = 0.1 has two arms; the upper arm corresponds to H^{**} increasing with γ up to its maximum value, and the lower arm to H^{**} decreasing with γ after reaching its maximum value.

Figure S4. The left-hand panel shows the mean pairwise diversities (relative to the purely neutral expectation) at a neutral site during the course of fixation of a completely linked deleterious mutation with dominance coefficient h = 0.1, for haplotypes carrying the ancestral allele (A_1), the new mutation (A_2), and between A_1 and A_2 haplotypes (A_1 versus

A_2). The final diversity at the time of fixation of A_2 and the mean diversity over all three haplotypes over the course of the fixation process are also shown. The statistics were estimated from simulations of a Wright-Fisher population with N = 50 by the method described in the text. The right-hand panel displays the mean values and standard errors of the net change in relative diversity over repeated fixation events for two different N values.

Figure S5. This figure shows the mean pairwise diversities (relative to the purely neutral expectation) at a neutral site during the course of loss of a completely linked selected mutation for haplotypes carrying the ancestral allele (A_1), the new mutation (A_2), and between A_1 and A_2 haplotypes (A_1 versus A_2). The final diversity at the time of fixation of A_2 and the mean diversity over all three haplotypes over the course of the fixation process are also shown. The left-hand panel shows the statistics for a favorable mutation with h = 0.9, and the right-hand panel for a deleterious mutation with h = 0.1.

Figures S6-S11. These show the various diversity statistics from Table S9 plotted against t^* or t^{**}, as appropriate. In Figures S6 and S9, the vertical blue lines indicate the values of t^* or t^{**} for $\gamma = 0$.
