# Supplementary material for: The effects of weak selection on neutral diversity at linked sites
Source: Genetics. 2022 Feb 12;221(1):iyac027. doi: 10.1093/genetics/iyac027 (PMC9071562; doi:10.1093/genetics/iyac027)
Supplement: iyac027_Supplementary_Data [file iyac027_supplementary_data.zip › Supplemental_File_1_GENETICS-2022-305040.pdf]

Supplementary File S1

S1.1 Analysis of the quadratic terms in Equations (2) - (5)

The quadratic terms in γ in Equations (2) and (3) are positive for biologically realistic values of a and b (see Box 1 of Charlesworth [2020a]), so that sufficiently strong selection causes t^* and t^{**} to decline with γ, as was found numerically by Mafessoni and Lachmann (2015) for the case of autosomal inheritance and random mating. Furthermore, the expressions for a and b in Box 1 of Charlesworth (2020a) show that, if the sign of γ is changed, t^* remains the same if the dominance coefficient h is changed to the complementary value of $1 - h$, generalizing the result of Maruyama and Kimura (1974).

This is not true of t^{**}, however. For the case of an autosomal mutation with random mating, the leading term in the multiplicand of $-\gamma^2/18$ in Equation (3) is equal to $h(7 - 4h)/2$. Substitution of $1 - h$ for h yields a multiplicand with a leading term of $(1 - h)(10 - 3h)/2$, and the difference between the two multiplicands is $[h(20 - 7h) - 10]/2$. If $h \leq 0.5$, this expression is always negative, implying that a weakly selected partially recessive deleterious mutation with dominance coefficient $h < 0.5$ will (somewhat paradoxically) experience a longer mean time to loss than the reverse favorable mutation with dominance coefficient $1 - h$.

In addition, H^* has the same properties as t^* with respective to the sign of γ and the complementary dominance coefficients h and $1 - h$, which follows from the fact that H^* differs from t^* only by a factor of $4N_ex(1 - x)$ when integrating its product with $t^*(x, q)$ with respect to x.

S1.2 Interpreting the determinants of the effect of fixations and losses on neutral diversity at a linked site

The results shown in Table S9 can be used to assess the relations between various diversity statistics and the sojourn times for a range of h values and two different $|\gamma|$ values, displayed in Figures S6-S11. Here, the results for $\gamma > 0$ and $\gamma < 0$ are shown in the same panels. For fixations, Figures S6-S8 show that $\Delta\pi_S$, $\Delta\pi_w$ and $-\Delta\pi_0$ are all nearly linearly related to t^* for a given $|\gamma|$ value, with $\Delta\pi_S$ and $\Delta\pi_w$ increasing with t^* and $-\Delta\pi_0$ decreasing with t^*. The plots for $\Delta\pi_S$ and $\Delta\pi_w$ are displaced downwards for the larger $|\gamma|$ value, whereas the plot for $-\Delta\pi_{0f}$ is displaced upwards. The reduction in diversity at the end of a fixation event ($-\Delta\pi_0$) is generally somewhat smaller for a given value of t^* for $\gamma < 0$ than for $\gamma > 0$, but a nearly linear relationship with t^* holds for a given value of γ.

Because t^{**} is different for mutations with a given $\gamma > 0$ and $\gamma < 0$ and dominance coefficients of h and $1 - h$, respectively, the pattern is more complex for loss events. The plots for $\Delta\pi_S$ and $\Delta\pi_w$ fall into two branches, with the left-hand branch for $\Delta\pi_S$ corresponding to $\gamma < 0$ and the right-hand branch $\gamma > 0$, and vice-versa for $\Delta\pi_w$. Within branches, the relations of $\Delta\pi_S$ and $\Delta\pi_w$ to t^{**} are similar to those for fixation events. Interestingly, the relation of t^{**} to $-\Delta\pi_0$ is in the opposite direction to that between of t^{**} to $-\Delta\pi_0$. For the stronger selection intensity, no division into two branches is seen for $-\Delta\pi_0$; for the weaker selection case, there is a very clear division, with the lower set corresponding to $\gamma < 0$.

Another complexity is revealed by the comparison of the results on fixation with $h = 0.9$ and $h = 0.1$ for a given value of $|\gamma|$ (Tables S6-S8). Especially for the larger values of $|\gamma|$, $\Delta\pi_S$ is larger for deleterious mutations with $h = 0.1$ than for favorable mutations with $h = 0.9$ and the same $|\gamma|$, even though t^* is the same in both cases. For example, $\Delta\pi_S = 0.0384 \pm 0.0036$ for $\gamma = -2$ and $h = 0.1$, but $\Delta\pi_S = 0.0325 \pm 0.0035$ for $\gamma = 2$ and $h = 0.9$; for $|\gamma| = 5$, the corresponding values of $\Delta\pi_S$ are – 0.200±0.003 and – 0.228±0.002, a nearly 30% difference.

S1.3 Interpreting the effects of selection on the conditional sojourn times

This approach uses conditional diffusion equations, which depend on the mean change in x for a given x (in units of $2N_e$ generations) for a trajectory conditioned on either fixation or loss (Ewens 2004, p.147; Zhao *et al.* 2015). Denoting the conditional mean changes in x for fixation and loss events by Δx^* and Δx^{**}, respectively, and using primes to denote derivatives, we have:

$$\Delta x^* = \Delta x + x(1-x)P_1'(x)P_1^{-1}(x) \qquad \text{(S1a)}$$

$$\Delta x^{**} = \Delta x + x(1-x)P_0'(x)P_0^{-1}(x) \qquad \text{(S1b)}$$

Using Equations (1), (A1) and (A2), these expressions can be approximated as follows, to an accuracy of first-order terms in γ:

$$\Delta x^* \approx (1-x)(1 + \tfrac{1}{3}\gamma b x^2) \qquad \text{(S2a)}$$

$$-\Delta x^{**} \approx x\{1 + \tfrac{1}{3}\gamma b[1 - x(2-x)]\} \qquad \text{(S2b)}$$

Consistent with the argument used above, there is no contribution from γa to these expressions. The terms added to Δx in these equations have been called "fictitious selection" by Zhao *et al*. (2015); here, these terms cancel the contribution from γa to Δx. In both cases, the multiplicand of the term in γb is positive for $0 < x < 1$, so that the tendencies for A_2 to increase in frequency relative to the neutral case, as measured by $\Delta x^{*}/(1-x)$, or to decrease in frequency, as measured by $-\Delta x^{**}/x$, are both reduced when $\gamma b < 0$, implying that the respective sojourn times are increased by selection.

Literature Cited

Charlesworth B. 2020a. How long does it take to fix a favorable mutation, and why should we care? Am. Nat. 195:753-771.

Charlesworth B. 2020b. How good are predictions of the effects of selective sweeps on levels of neutral diversity? Genetics 216:1217-1239.

Ewens WJ. 2004. Mathematical Population Genetics. 1. Theoretical Introduction. New York: Springer.

Mafessoni F, Lachmann D. 2015. Selective strolls: fixation and extinction in diploids are slower for weakly selected mutations than for neutral ones. Genetics 201:1581-1589.

Maruyama T, Kimura M. 1974. A note on the speed of gene frequency changes in reverse directions in a finite population. Evolution 28:161-163.

Zhao L, Lascoux M, Overall ADJ, Waxman D. 2015. The characteristic trajectory of a fixing allele: A consequence of fictitious selection that arises from conditioning. Genetics 195:993-1006
