# Supplementary material for: The effects of weak selection on neutral diversity at linked sites
Source: Genetics. 2022 Feb 12;221(1):iyac027. doi: 10.1093/genetics/iyac027 (PMC9071562; doi:10.1093/genetics/iyac027)
Supplement: iyac027_Supplementary_Data [file iyac027_supplementary_data.zip › Supplemental_Table_1_GENETICS-2022-305040.docx]

**Table S1. Fixations of favorable mutations with *h* = 0.9 and no recombination**

**(times are in units of 2*N* generations; diversities are relative to the equilibrium value with no selection)**

**Population size= 50**

**Number of replicate fixations= 100000**

**Initial A2 allele frequency= 9.99999978E-03**

**gamma= 0.00000000**

Total number of runs= 9987096

Frequency of fixations of A2= 1.00129209E-02

Mean time to fixation= 1.96752441 s.e.= 3.38354707E-03

Mean weighted relative diversities over paths to fixation

A1A1= 0.616873085 s.e.= 9.12600837E-04

A1A2= 2.27963734 s.e.= 6.90885168E-03

A2A2= 0.327654451 s.e.= 7.91461149E-04

Mean= 1.20851672 s.e.= 3.22370674E-03

Mean final relative diversity= 0.572995067 s.e.= 3.37116624E-04

Mean final diversity reduction= 0.427004933 s.e.= 3.37116624E-04

Weighted measure of potential recurrent sweep effect= -1.67397149E-02

s.e.= 3.25853680E-03

**gamma= 0.500000000**

Total number of runs= 7383439

Frequency of fixations of A2= 1.35438247E-02

Mean time to fixation= 2.00696802 s.e.= 3.48993600E-03

Mean weighted relative diversities over paths to fixation

A1A1= 0.614189148 s.e.= 9.21358995E-04

A1A2= 2.31189060 s.e.= 7.14778760E-03

A2A2= 0.329604208 s.e.= 7.97818182E-04

Mean= 1.22070241 s.e.= 3.32239713E-03

Mean final relative diversity= 0.574947596 s.e.= 3.37913487E-04

Mean final diversity reduction= 0.425052404 s.e.= 3.37913487E-04

Weighted measure of potential recurrent sweep effect= 1.79079156E-02

s.e.= 3.49537260E-03

**gamma= 1.00000000**

Total number of runs= 5606348

Frequency of fixations of A2= 1.78369228E-02

Mean time to fixation = 2.02606201 s.e.= 3.55402404E-03

Mean weighted relative diversities over paths to fixation

A1A1= 0.611352682 s.e.= 9.19748971E-04

A1A2= 2.32971144 s.e.= 7.22233159E-03

A2A2= 0.331986666 s.e.= 8.10938305E-04

Mean= 1.22748411 s.e.= 3.35339154E-03

Mean final relative diversity= 0.576064944 s.e.= 3.39255814E-04

Mean final diversity reduction= 0.423935056 s.e.= 3.39255814E-04

Weighted measure of potential recurrent sweep effect= 3.69526483E-02

s.e.= 3.54766962E-03

**gamma= 1.50000000**

Total number of runs= 4304958

Frequency of fixations of A2= 2.32290309E-02

Mean time to fixation = 2.03380847 s.e.= 3.57047189E-03

Mean weighted relative diversities over paths to fixation

A1A1= 0.609204710 s.e.= 9.12200834E-04

A1A2= 2.33526564 s.e.= 7.24367378E-03

A2A2= 0.333696812 s.e.= 8.17897613E-04

Mean= 1.22965515 s.e.= 3.36397719E-03

Mean final relative diversity= 0.577155054 s.e.= 3.39002989E-04

Mean final diversity reduction= 0.422844946 s.e.= 3.39002989E-04

Weighted measure of potential recurrent sweep effect= 4.42213379E-02

s.e.= 3.57948104E-03

**gamma= 2.00000000**

Total number of runs= 3415218

Frequency of fixations of A2= 2.92807072E-02

Mean time to fixation= 2.02294731 s.e.= 3.53671587E-03

Mean weighted relative diversities over paths to fixation

A1A1= 0.608090937 s.e.= 9.05710971E-04

A1A2= 2.32557559 s.e.= 7.15426449E-03

A2A2= 0.334168732 s.e.= 8.17680615E-04

Mean= 1.22481287 s.e.= 3.32351774E-03

Mean final relative diversity= 0.577659369 s.e.= 3.39256396E-04

Mean final diversity reduction= 0.422340631 s.e.= 3.39256396E-04

Weighted measure of potential recurrent sweep effect= 3.24577875E-02

s.e.= 3.49148549E-03

**gamma= 2.50000000**

Total number of runs= 2775606

Frequency of fixations of A2= 3.60281691E-02

Mean time to fixation= 1.98594999 s.e.= 3.43233580E-03

Mean weighted relative diversities over paths to fixation

A1A1= 0.607519805 s.e.= 8.88933660E-04

A1A2= 2.29451466 s.e.= 6.97115250E-03

A2A2= 0.334696919 s.e.= 8.18807981E-04

Mean= 1.21225357 s.e.= 3.24832415E-03

Mean final relative diversity= 0.577090621 s.e.= 3.40605475E-04

Mean final diversity reduction= 0.422909379 s.e.= 3.40605475E-04

Weighted measure of potential recurrent sweep effect= -1.37208996E-03

s.e.= 3.32386885E-03

**gamma= 3.00000000**

Total number of runs= 2328547

Frequency of fixations of A2= 4.29452360E-02

Mean time to fixation (2N generation units)= 1.95147872 s.e.= 3.37429252E-03

Mean weighted relative diversities over paths to fixation

A1A1= 0.606406868 s.e.= 8.82535940E-04

A1A2= 2.27242446 s.e.= 6.89876825E-03

A2A2= 0.335140139 s.e.= 8.26491450E-04

Mean= 1.20239878 s.e.= 3.22069763E-03

Mean final relative diversity= 0.576430678 s.e.= 3.42130254E-04

Mean final diversity reduction= 0.423569322 s.e.= 3.42130254E-04

Weighted measure of potential recurrent sweep effect= -2.85685007E-02

s.e.= 3.21760168E-03

**gamma= 3.50000000**

Total number of runs= 1970779

Frequency of fixations of A2= 5.07413559E-02

Mean time to fixation= 1.90122163 s.e.= 3.21284262E-03

Mean weighted relative diversities over paths to fixation

A1A1= 0.605788231 s.e.= 8.58215964E-04

A1A2= 2.22703147 s.e.= 6.54750876E-03

A2A2= 0.335123271 s.e.= 8.17876018E-04

Mean= 1.18282032 s.e.= 3.06557608E-03

Mean final relative diversity= 0.576127231 s.e.= 3.44248518E-04

Mean final diversity reduction= 0.423872769 s.e.= 3.44248518E-04

Weighted measure of potential recurrent sweep effect= -7.62951225E-02

s.e.= 2.92380666E-03

**gamma= 4.00000000**

Total number of runs= 1704656

Frequency of fixations of A2= 5.86628616E-02

Mean time to fixation= 1.84227788 s.e.= 3.07030231E-03

Mean weighted relative diversities over paths to fixation

A1A1= 0.606282473 s.e.= 8.38744687E-04

A1A2= 2.18192840 s.e.= 6.29458390E-03

A2A2= 0.333954543 s.e.= 8.18245346E-04

Mean= 1.16365051 s.e.= 2.95495824E-03

Mean final relative diversity= 0.574033856 s.e.= 3.44495813E-04

Mean final diversity reduction= 0.425966144 s.e.= 3.44495813E-04

Weighted measure of potential recurrent sweep effect= -0.124448188

s.e.= 2.67390069E-03

**gamma= 4.50000000**

Total number of runs= 1493965

Frequency of fixations of A2= 6.69359714E-02

Mean time to fixation= 1.77671325 s.e.= 2.90295691E-03

Mean weighted relative diversities over paths to fixation

A1A1= 0.606995046 s.e.= 8.18767876E-04

A1A2= 2.13046074 s.e.= 5.93078043E-03

A2A2= 0.332499892 s.e.= 8.10824684E-04

Mean= 1.14162195 s.e.= 2.79632513E-03

Mean final relative diversity= 0.571604848 s.e.= 3.47798952E-04

Mean final diversity reduction= 0.428395152 s.e.= 3.47798952E-04

Weighted measure of potential recurrent sweep effect= -0.176792488

s.e.= 2.35822471E-03

**gamma= 5.00000000**

Total number of runs= 1332801

Frequency of fixations of A2= 7.50299543E-02

Mean time to fixation= 1.70737600 s.e.= 2.73282733E-03

Predicted approximate mean time to fixation= 1.84479642

Mean weighted relative diversities over paths to fixation

A1A1= 0.608333290 s.e.= 7.99194211E-04

A1A2= 2.07731056 s.e.= 5.62612806E-03

A2A2= 0.330334932 s.e.= 8.01159942E-04

Mean= 1.11898017 s.e.= 2.66742823E-03

Mean final relative diversity= 0.568600059 s.e.= 3.49862210E-04

Mean final diversity reduction= 0.431399941 s.e.= 3.49862210E-04

Weighted measure of potential recurrent sweep effect= -0.228240952

s.e.= 2.11777096E-03
