# Supplementary material for: The effects of weak selection on neutral diversity at linked sites
Source: Genetics. 2022 Feb 12;221(1):iyac027. doi: 10.1093/genetics/iyac027 (PMC9071562; doi:10.1093/genetics/iyac027)
Supplement: iyac027_Supplementary_Data [file iyac027_supplementary_data.zip › Supplemental_Table_2_GENETICS-2022-305040.docx]

**Table S2. Fixations of deleterious mutations with *h* = 0.1 and no recombination (times are in units of 2*N* generations; diversities are relative to the equilibrium value with no selection)**

**Population size= 50**

**Number of replicate fixations= 100000**

**Initial A2 allele frequency= 9.99999978E-03**

**gamma= 0.00000000**

Total number of runs= 9987096

Frequency of fixations of A2= 1.00129209E-02

Mean time to fixation= 1.96752441 s.e.= 3.38354707E-03

Mean weighted relative diversities over paths to fixation

A1A1= 0.616873085 s.e.= 9.12600837E-04

A1A2= 2.27963734 s.e.= 6.90885168E-03

A2A2= 0.327654451 s.e.= 7.91461149E-04

Mean= 1.20851672 s.e.= 3.22370674E-03

Mean final relative diversity= 0.572995067 s.e.= 3.37116624E-04

Mean final diversity reduction= 0.427004933 s.e.= 3.37116624E-04

Weighted measure of potential recurrent sweep effect= -1.67397149E-02

s.e.= 3.25853680E-03

**gamma= –0.500000000**

Total number of runs= 12200123

Frequency of fixations of A2= 8.19663890E-03

Mean time to fixation= 1.99705887 s.e.= 3.47644440E-03

Mean weighted relative diversities over paths to fixation

A1A1= 0.615017116 s.e.= 9.21456376E-04

A1A2= 2.30606413 s.e.= 7.07197888E-03

A2A2= 0.328970999 s.e.= 7.98719877E-04

Mean= 1.21916926 s.e.= 3.29825934E-03

Mean final relative diversity= 0.573821723 s.e.= 3.36557074E-04

Mean final diversity reduction= 0.426178277 s.e.= 3.36557074E-04

Weighted measure of potential recurrent sweep effect= 1.15009323E-02

s.e.= 3.40887764E-03

**gamma= -1.00000000**

Total number of runs= 15061308

Frequency of fixations of A2= 6.63952949E-03

Mean time to fixation= 2.02778482 s.e.= 3.54683376E-03

Mean weighted relative diversities over paths to fixation

A1A1= 0.613901734 s.e.= 9.22767213E-04

A1A2= 2.32904983 s.e.= 7.19050597E-03

A2A2= 0.330081016 s.e.= 8.04053270E-04

Mean= 1.22826505 s.e.= 3.34826810E-03

Mean final relative diversity= 0.574972332 s.e.= 3.37336154E-04

Mean final diversity reduction= 0.425027668 s.e.= 3.37336154E-04

Weighted measure of potential recurrent sweep effect= 3.78471985E-02

s.e.= 3.54993553E-03

**gamma= -1.50000000**

Total number of runs= 19080277

Frequency of fixations of A2= 5.24101453E-03

Mean time to fixation= 2.02818370 s.e.= 3.54042673E-03

Mean weighted relative diversities over paths to fixation

A1A1= 0.615842760 s.e.= 9.29849746E-04

A1A2= 2.32807302 s.e.= 7.17790751E-03

A2A2= 0.328471988 s.e.= 7.94499123E-04

Mean= 1.22789955 s.e.= 3.33715533E-03

Mean final relative diversity= 0.574273348 s.e.= 3.36373079E-04

Mean final diversity reduction= 0.425726652 s.e.= 3.36373079E-04

Weighted measure of potential recurrent sweep effect= 3.65039445E-02

s.e.= 3.52822873E-03

**gamma= -2.00000000**

Total number of runs= 24679659

Frequency of fixations of A2= 4.05191956E-03

Mean time to fixation= 2.02160358 s.e.= 3.57255945E-03

Mean weighted relative diversities over paths to fixation

A1A1= 0.617767155 s.e.= 9.41565202E-04

A1A2= 2.33140159 s.e.= 7.31596816E-03

A2A2= 0.327065885 s.e.= 8.00585956E-04

Mean= 1.23077595 s.e.= 3.40943015E-03

Mean final relative diversity= 0.571886539 s.e.= 3.36586381E-04

Mean final diversity reduction= 0.428113461 s.e.= 3.36586381E-04

Weighted measure of potential recurrent sweep effect= 3.84122580E-02

s.e.= 3.62818572E-03

**gamma= -2.50000000**

Total number of runs= 32720937

Frequency of fixations of A2= 3.05614737E-03

Mean time to fixation = 1.99342620 s.e.= 3.47175682E-03

Mean weighted relative diversities over paths to fixation

A1A1= 0.622755408 s.e.= 9.43856547E-04

A1A2= 2.30397463 s.e.= 7.06717605E-03

A2A2= 0.323284090 s.e.= 7.76533445E-04

Mean= 1.22085583 s.e.= 3.30313714E-03

Mean final relative diversity= 0.569398403 s.e.= 3.35312274E-04

Mean final diversity reduction= 0.430601597 s.e.= 3.35312274E-04

Weighted measure of potential recurrent sweep effect= 9.63399652E-03

s.e.= 3.41310236E-03

**gamma= -3.00000000**

Total number of runs= 44394161

Frequency of fixations of A2= 2.25254847E-03

Mean time to fixation= 1.96174359 s.e.= 3.38393892E-03

Mean weighted relative diversities over paths to fixation

A1A1= 0.628218055 s.e.= 9.49337613E-04

A1A2= 2.27769184 s.e.= 6.90173637E-03

A2A2= 0.318799853 s.e.= 7.58091745E-04

Mean= 1.21121538 s.e.= 3.23359575E-03

Mean final relative diversity= 0.566026866 s.e.= 3.32383293E-04

Mean final diversity reduction= 0.433973134 s.e.= 3.32383293E-04

Weighted measure of potential recurrent sweep effect= -1.96198914E-02

s.e.= 3.25265387E-03

**gamma= -3.50000000**

Total number of runs= 60646912

Frequency of fixations of A2= 1.64888857E-03

Mean time to fixation= 1.90945339 s.e.= 3.23752081E-03

Mean weighted relative diversities over paths to fixation

A1A1= 0.634604156 s.e.= 9.51195136E-04

A1A2= 2.23414779 s.e.= 6.55922573E-03

A2A2= 0.313900739 s.e.= 7.36763352E-04

Mean= 1.19497991 s.e.= 3.09035019E-03

Mean final relative diversity= 0.561538637 s.e.= 3.31963151E-04

Mean final diversity reduction= 0.438461363 s.e.= 3.31963151E-04

Weighted measure of potential recurrent sweep effect= -6.61685914E-02

s.e.= 2.94053974E-03

**gamma= -4.00000000**

Total number of runs= 84419953

Frequency of fixations of A2= 1.18455407E-03

Mean time to fixation= 1.85586917 s.e.= 3.10254516E-03

Mean weighted relative diversities over paths to fixation

A1A1= 0.640763044 s.e.= 9.53360402E-04

A1A2= 2.19223309 s.e.= 6.29896484E-03

A2A2= 0.308944225 s.e.= 7.16474548E-04

Mean= 1.17919159 s.e.= 2.98601598E-03

Mean final relative diversity= 0.556841195 s.e.= 3.30784911E-04

Mean final diversity reduction= 0.443158805 s.e.= 3.30784911E-04

Weighted measure of potential recurrent sweep effect= -0.110589027

s.e.= 2.70965486E-03

**gamma= -4.50000000**

Total number of runs= 118542323

Frequency of fixations of A2= 8.43580579E-04

Mean time to fixation= 1.80200779 s.e.= 2.95959110E-03

A1A1= 0.649086416 s.e.= 9.56025964E-04

A1A2= 2.14898419 s.e.= 5.99469012E-03

A2A2= 0.302532494 s.e.= 6.90546061E-04

Mean= 1.16360843 s.e.= 2.86033587E-03

Mean final relative diversity= 0.551111221 s.e.= 3.28066206E-04

Mean final diversity reduction= 0.448888779 s.e.= 3.28066206E-04

Weighted measure of potential recurrent sweep effect= -0.154078886

s.e.= 2.45507644E-03

**gamma= -5.00000000**

Total number of runs= 170624788

Frequency of fixations of A2= 5.86081354E-04

Mean time to fixation= 1.74085200 s.e.= 2.81718443E-03

Mean weighted relative diversities over paths to fixation

A1A1= 0.656760216 s.e.= 9.64585692E-04

A1A2= 2.10328817 s.e.= 5.76179614E-03

A2A2= 0.296419472 s.e.= 6.66680920E-04

Mean= 1.14655852 s.e.= 2.76617333E-03

Mean final relative diversity= 0.545240521 s.e.= 3.25853267E-04

Mean final diversity reduction= 0.454759479 s.e.= 3.25853267E-04

Weighted measure of potential recurrent sweep effect= -0.199634120

s.e.= 2.25348561E-03
