# Supplementary material for: The effects of weak selection on neutral diversity at linked sites
Source: Genetics. 2022 Feb 12;221(1):iyac027. doi: 10.1093/genetics/iyac027 (PMC9071562; doi:10.1093/genetics/iyac027)
Supplement: iyac027_Supplementary_Data [file iyac027_supplementary_data.zip › Supplemental_Table_3_GENETICS-2022-305040.docx]

**Table S3. Fixations of favorable mutations with *h* = 0.9 and no recombination**

**(times are in units of 2*N* generations; diversities are relative to the equilibrium value with no selection)**

**Population size= 500**

**Number of replicate fixations= 10000**

**Initial A2 allele frequency= 1.00000005E-03**

**gamma= 0.00000000**

Total number of runs= 9918878

Frequency of fixations of A2= 1.00817857E-03

Mean time to fixation= 1.98005831 s.e.= 1.06094023E-02

Mean weighted relative diversities over paths to fixation

A1A1= 0.617362261 s.e.= 2.85766809E-03

A1A2= s.e.= 2.14616098E-02

A2A2= 0.325654536 s.e.= 2.45830952E-03

Mean= 1.20560277 s.e.= 1.00571467E-02

Mean final relative diversity= 0.577506602 s.e.= 1.06250599E-03

Mean final diversity reduction= 0.422493398 s.e.= 1.06250599E-03

Weighted measure of potential recurrent sweep effect= -1.53915137E-02

s.e.= 1.02540981E-02

**gamma= 0.500000000**

Total number of runs= 7201848

Frequency of fixations of A2= 1.38853246E-03

Mean time to fixation= 2.01161909 s.e.= 1.07705407E-02

Mean weighted relative diversities over paths to fixation

A1A1= 0.615959287 s.e.= 2.85740057E-03

A1A2= 2.29459715 s.e.= 2.15229969E-02

A2A2= 0.327493608 s.e.= 2.47753831E-03

Mean= 1.21257603 s.e.= 1.00137405E-02

Mean final relative diversity= 0.579937577 s.e.= 1.06093881E-03

Mean final diversity reduction= 0.420062423 s.e.= 1.06093881E-03

Weighted measure of potential recurrent sweep effect= 7.55587360E-03

s.e.= 1.02810394E-02

**gamma= 1.00000000**

Total number of runs= 5556515

Frequency of fixations of A2= 1.79968914E-03

Mean time to fixation= 2.06010795 s.e.= 1.12161171E-02

Mean weighted relative diversities over paths to fixation

A1A1= 0.611150324 s.e.= 2.87304190E-03

A1A2= 2.33583450 s.e.= 2.26326976E-02

A2A2= 0.330684841 s.e.= 2.51130643E-03

Mean= 1.22906113 s.e.= 1.05327899E-02

Mean final relative diversity= 0.582387984 s.e.= 1.05781457E-03

Mean final diversity reduction= 0.417612016 s.e.= 1.05781457E-03

Weighted measure of potential recurrent sweep effect= 5.42744175E-02

s.e.= 1.14496313E-02

**gamma= 1.50000000**

Total number of runs= 4331338

Frequency of fixations of A2= 2.30875541E-03

Mean time to fixation= 2.07283139 s.e.= 1.13679217E-02

Predicted approximate mean time to fixation= 2.07937241

Mean weighted relative diversities over paths to fixation

A1A1= 0.610856950 s.e.= 2.89083994E-03

A1A2= 2.34859085 s.e.= 2.28973217E-02

A2A2= 0.331399411 s.e.= 2.52840412E-03

Mean= 1.23390388 s.e.= 1.06378645E-02

Mean final relative diversity= 0.583700657 s.e.= 1.06354989E-03

Mean final diversity reduction= 0.416299343 s.e.= 1.06354989E-03

Weighted measure of potential recurrent sweep effect= 6.85371161E-02

s.e.= 1.16508035E-02

**gamma= 2.00000000**

Total number of runs= 3380315

Frequency of fixations of A2= 2.95830425E-03

Mean time to fixation= 2.05545616 s.e.= 1.12903183E-02

Mean weighted relative diversities over paths to fixation

A1A1= 0.608085334 s.e.= 2.84519326E-03

A1A2= 2.33825731 s.e.= 2.28275713E-02

A2A2= 0.333779007 s.e.= 2.58594425E-03

Mean= 1.22857368 s.e.= 1.05544301E-02

Mean final relative diversity= 0.582768917 s.e.= 1.07046298E-03

Mean final diversity reduction= 0.417231083 s.e.= 1.07046298E-03

Weighted measure of potential recurrent sweep effect= 5.25954887E-02

s.e.= 1.13744447E-02

**gamma= 2.50000000**

Total number of runs= 2726919

Frequency of fixations of A2= 3.66714224E-03

Mean time to fixation= 2.03389883 s.e.= 1.12186624E-02

Mean weighted relative diversities over paths to fixation

A1A1= 0.606070578 s.e.= 2.85001658E-03

A1A2= 2.32680607 s.e.= 2.27098484E-02

A2A2= 0.334668487 s.e.= 2.59734108E-03

Mean= 1.22271264 s.e.= 1.05369417E-02

Mean final relative diversity= 0.583613217 s.e.= c

Mean final diversity reduction= 0.416386783 s.e.= 1.06887543E-03

Weighted measure of potential recurrent sweep effect= 3.65930386E-02

s.e.= 1.11754658E-02

**gamma= 3.00000000**

Total number of runs= 2296863

Frequency of fixations of A2= 4.35376447E-03

Mean time to fixation= 2.00179267 s.e.= 1.11313853E-02

Mean weighted relative diversities over paths to fixation

A1A1= 0.605677605 s.e.= 2.83204624E-03

A1A2= 2.31083322 s.e.= 2.34213695E-02

A2A2= 0.334649950 s.e.= 2.66186683E-03

Mean= 1.21449232 s.e.= 1.07509773E-02

Mean final relative diversity= 0.582821965 s.e.= 1.09056314E-03

Mean final diversity reduction= 0.417178035 s.e.= 1.09056314E-03

Weighted measure of potential recurrent sweep effect= 1.21871578E-02

s.e.= 1.14644067E-02

**gamma= 3.50000000**

Total number of runs= 1893159

Frequency of fixations of A2= 5.28217666E-03

Mean time to fixation= 1.94483840 s.e.= 1.03110876E-02

Predicted approximate mean time to fixation= 2.01729107

Mean weighted relative diversities over paths to fixation

A1A1= 0.607401371 s.e.= 2.73512769E-03

A1A2= 2.24621749 s.e.= 2.05694679E-02

A2A2= 0.332242221 s.e.= 2.52313958E-03

Mean= 1.18933165 s.e.= 9.60476603E-03

Mean final relative diversity= 0.581397951 s.e.= 1.07124960E-03

Mean final diversity reduction= 0.418602049 s.e.= 1.07124960E-03

Weighted measure of potential recurrent sweep effect= -5.03756069E-02

s.e.= 9.25712381E-03

**gamma= 4.00000000**

Total number of runs= 1646251

Frequency of fixations of A2= 6.07440807E-03

Mean time to fixation= 1.88398278 s.e.= 9.99999139E-03

Mean weighted relative diversities over paths to fixation

A1A1= 0.606749833 s.e.= 2.67213723E-03

A1A2= 2.20784783 s.e.= 2.07472555E-02

A2A2= 0.332965583 s.e.= 2.60009826E-03

Mean= 1.17334938 s.e.= 9.66125075E-03

Mean final relative diversity= 0.580359221 s.e.= 1.07335020E-03

Mean final diversity reduction= 0.419640779 s.e.= 1.07335020E-03

Weighted measure of potential recurrent sweep effect= -9.30588022E-02

s.e.= 9.19064973E-03

**gamma= 4.50000000**

Total number of runs= 1463044

Frequency of fixations of A2= 6.83506439E-03

Mean time to fixation= 1.84750843 s.e.= 9.68233682E-03

Mean weighted relative diversities over paths to fixation

A1A1= 0.605997205 s.e.= 2.64160521E-03

A1A2= 2.17793751 s.e.= 2.02643927E-02

A2A2= 0.332592934 s.e.= 2.57893209E-03

Mean= 1.15983355 s.e.= 9.48380027E-03

Mean final relative diversity= 0.579967320 s.e.= 1.07774173E-03

Mean final diversity reduction= 0.420032680 s.e.= 1.07774173E-03

Weighted measure of potential recurrent sweep effect= -0.124749474

s.e.= 8.83596577E-03

**gamma= 5.00000000**

Total number of runs= 1317422

Frequency of fixations of A2= 7.59058213E-03

Mean time to fixation= 1.76234639 s.e.= 8.87480471E-03

Mean weighted relative diversities over paths to fixation

A1A1= 0.607972682 s.e.= 2.52312911E-03

A1A2= 2.10509872 s.e.= 1.80586372E-02

A2A2= 0.329556257 s.e.= 2.51930673E-03

Mean= 1.12850642 s.e.= 8.47924780E-03

Mean final relative diversity= 0.574976325 s.e.= 1.09096267E-03

Mean final diversity reduction= 0.425023675 s.e.= 1.09096267E-03

Weighted measure of potential recurrent sweep effect= -0.198551714

s.e.= 6.99326163E-03
