# Supplementary material for: The effects of weak selection on neutral diversity at linked sites
Source: Genetics. 2022 Feb 12;221(1):iyac027. doi: 10.1093/genetics/iyac027 (PMC9071562; doi:10.1093/genetics/iyac027)
Supplement: iyac027_Supplementary_Data [file iyac027_supplementary_data.zip › Supplemental_Table_4_GENETICS-2022-305040.docx]

**Table S4. Fixations of deleterious mutations with *h* = 0.1 and no recombination(times are in units of 2*N* generations; diversities are relative to the equilibrium value with no selection)**

**Population size= 500**

**Number of replicate fixations= 10000**

**Initial A2 allele frequency= 1.00000005E-03**

**gamma= -0.500000000**

Total number of runs= 12346012

Frequency of fixations of A2= 8.09978170E-04

Mean time to fixation= 2.03531694 s.e.= 1.12439273E-02

Mean weighted relative diversities over paths to fixation

A1A1= 0.615276814 s.e.= 2.93674436E-03

A1A2= 2.32869148 s.e.= 2.25497242E-02

A2A2= 0.327481002 s.e.= 2.52617639E-03

Mean= 1.22706258 s.e.= 1.05175180E-02

Mean final relative diversity= 0.578265429 s.e.= 1.06679345E-03

Mean final diversity reduction= 0.421734571 s.e.= 1.06679345E-03

Weighted measure of potential recurrent sweep effect= 4.04103175E-02

s.e.= 1.10841477E-02

**gamma= -1.00000000**

Total number of runs= 15213606

Frequency of fixations of A2= 6.57306344E-04

Mean time to fixation= 2.05219150 s.e.= 1.12902941E-02

Mean weighted relative diversities over paths to fixation

A1A1= 0.615584850 s.e.= 2.92822439E-03

A1A2= 2.33711600 s.e.= 2.29473338E-02

A2A2= 0.327572286 s.e.= 2.51056533E-03

Mean= 1.23065782 s.e.= 1.06429365E-02

Mean final relative diversity= 0.579881847 s.e.= 1.05044420E-03

Mean final diversity reduction= 0.420118153 s.e.= 1.05044420E-03

Weighted measure of potential recurrent sweep effect= 5.32271639E-02

s.e.= 1.15076285E-02

**gamma= -1.50000000**

Total number of runs= 19263727

Frequency of fixations of A2= 5.19110297E-04

Mean time to fixation= 2.06498051 s.e.= 1.15180304E-02

Mean weighted relative diversities over paths to fixation

A1A1= 0.614455342 s.e.= 2.95709935E-03

A1A2= 2.35416579 s.e.= 2.38354281E-02

A2A2= 0.328419358 s.e.= 2.54808785E-03

Mean= 1.23697460 s.e.= 1.10195931E-02

Mean final relative diversity= 0.580740333 s.e.= 1.04992697E-03

Mean final diversity reduction= 0.419259667 s.e.= 1.04992697E-03

Weighted measure of potential recurrent sweep effect= 7.00905845E-02

s.e.= 1.22318892E-02

**gamma= -2.00000000**

Total number of runs= 24880156

Frequency of fixations of A2= 4.01926751E-04

Mean time to fixation= 2.04615045 s.e.= 1.11957965E-02

Mean weighted relative diversities over paths to fixation

A1A1= 0.615954161 s.e.= 2.91022519E-03

A1A2= 2.32981801 s.e.= 2.28568912E-02

A2A2= 0.327787876 s.e.= 2.50264211E-03

Mean= 1.22900951 s.e.= 1.06502241E-02

Mean final relative diversity= 0.580353439 s.e.= 1.04003865E-03

Mean final diversity reduction= 0.419646561 s.e.= 1.04003865E-03

Weighted measure of potential recurrent sweep effect= 4.89528067E-02

s.e.= 1.15499934E-02

**gamma= -2.50000000**

Total number of runs= 33613773

Frequency of fixations of A2= 2.97497114E-04

Mean time to fixation= 2.03471327 s.e.= 1.11346459E-02

Mean weighted relative diversities over paths to fixation

A1A1= 0.623357654 s.e.= 2.97903945E-03

A1A2= 2.32247496 s.e.= 2.23819278E-02

A2A2= 0.321503580 s.e.= 2.43970752E-03

Mean= 1.22538948 s.e.= 1.03912652E-02

Mean final relative diversity= 0.575192750 s.e.= 1.05385098E-03

Mean final diversity reduction= 0.424807250 s.e.= 1.05385098E-03

Weighted measure of potential recurrent sweep effect= 3.37951891E-02

s.e.= 1.09377308E-02

**gamma= -3.00000000**

Total number of runs= 45302187

Frequency of fixations of A2= 2.20739894E-04

Mean time to fixation= 2.00417542 s.e.= 1.07443379E-02

Mean weighted relative diversities over paths to fixation

A1A1= 0.625449479 s.e.= 2.92414543E-03

A1A2= 2.29053617 s.e.= 2.17546448E-02

A2A2= 0.319433719 s.e.= 2.39041983E-03

Mean= 1.21246469 s.e.= 1.01331938E-02

Mean final relative diversity= 0.575368524 s.e.= 1.04457384E-03

Mean final diversity reduction= 0.424631476 s.e.= 1.04457384E-03

Weighted measure of potential recurrent sweep effect= 1.18363334E-03

s.e.= 1.04985749E-02

**gamma= -3.50000000**

Total number of runs= 6227130

Frequency of fixations of A2= 1.60587617E-04

Mean time to fixation= 1.93813598 s.e.= 3.15188281E-02

Mean weighted relative diversities over paths to fixation

A1A1= 0.635107279 s.e.= 9.03133862E-03

A1A2= 2.22559237 s.e.= 6.36206642E-02

A2A2= 0.314652711 s.e.= 7.52442423E-03

Mean= 1.19128525 s.e.= 2.98694763E-02

Mean final relative diversity= 0.569535255 s.e.= 3.28149577E-03

Mean final diversity reduction= 0.430464745 s.e.= 3.28149577E-03

Weighted measure of potential recurrent sweep effect= -5.97276539E-02

s.e.= 2.91576106E-02

**gamma= -4.00000000**

Total number of runs= 8629686

Frequency of fixations of A2= 1.15879069E-04

Mean time to fixation= 1.89929390 s.e.= 3.29806246E-02

Mean weighted relative diversities over paths to fixation

A1A1= 0.640802860 s.e.= 9.99871455E-03

A1A2= 2.23620081 s.e.= 6.86131045E-02

A2A2= 0.306685716 s.e.= 7.27875018E-03

Mean= 1.19497204 s.e.= 3.24524194E-02

Mean final relative diversity= 0.561796427 s.e.= 3.21675395E-03

Mean final diversity reduction= 0.438203573 s.e.= 3.21675395E-03

Weighted measure of potential recurrent sweep effect= -6.78945482E-02

s.e.= 3.14241871E-02

**gamma= -4.50000000**

Total number of runs= 12829435

Frequency of fixations of A2= 7.79457550E-05

Mean time to fixation= 1.84852791 s.e.= 3.15219052E-02

Mean weighted relative diversities over paths to fixation

A1A1= 0.641308248 s.e.= 9.72098112E-03

A1A2= 2.19325185 s.e.= 6.28633872E-02

A2A2= 0.307046741 s.e.= 7.40838284E-03

Mean= 1.17488563 s.e.= 2.95868367E-02

Mean final relative diversity= 0.561004698 s.e.= 3.37543106E-03

Mean final diversity reduction= 0.438995302 s.e.= 3.37543106E-03

Weighted measure of potential recurrent sweep effect= -0.115715235

s.e.= 2.58128867E-02

**gamma= -5.00000000**

Total number of runs= 18105553

Frequency of fixations of A2= 5.52316778E-05

Mean time to fixation= 1.74561203 s.e.= 2.85308305E-02

Mean weighted relative diversities over paths to fixation

A1A1= 0.655604601 s.e.= 9.77614336E-03

A1A2= 2.10623074 s.e.= 5.82417846E-02

A2A2= 0.294413835 s.e.= 6.64729020E-03

Mean= 1.14518631 s.e.= 2.78589185E-02

Mean final relative diversity= 0.550689578 s.e.= 3.19661992E-03

Mean final diversity reduction= 0.449310422 s.e.= 3.19661992E-03

Weighted measure of potential recurrent sweep effect= -0.195872441

s.e.= 2.25129705E-02
