# Supplementary material for: The effects of weak selection on neutral diversity at linked sites
Source: Genetics. 2022 Feb 12;221(1):iyac027. doi: 10.1093/genetics/iyac027 (PMC9071562; doi:10.1093/genetics/iyac027)
Supplement: iyac027_Supplementary_Data [file iyac027_supplementary_data.zip › Supplemental_Table_5_GENETICS-2022-305040.docx]

**Table S5. Losses of favorable mutations with *h* = 0.9 and no recombination**

**(times are in units of 2*N* generations; diversities are relative to the equilibrium value with no selection)**

**Population size= 50**

**Number of replicate fixations= 1000000**

**Initial A2 allele frequency= 1.00000005E-02**

**gamma= 0.500000**

Total number of runs= 1013472

Frequency of losses of A2= 0.986707

Mean time to loss= 8.833157E-02 s.e.= 2.690816E-04

Mean weighted relative diversities over paths to loss

A1A1= 0.922475 s.e.= 2.347653E-03

A1A2= 1.45675 s.e.= 7.808037E-03

A2A2= 9.153422E-02 s.e.= 7.743558E-04

Mean= 1.06962 s.e.= 4.078378E-03

Mean final relative diversity= 0.994735 s.e.= 2.206667E-05

Mean final diversity reduction= 5.265117E-03 s.e.= 2.206667E-05

Weighted measure of potential recurrent sweep effect= 2.099865E-05

s.e.= 1.066120E-04

**gamma= 1.00000**

Total number of runs= 1017731

Frequency of losses of A2= 0.982578

Mean time to loss= 8.833721E-02 s.e.= 2.708262E-04

Mean weighted relative diversities over paths to loss

A1A1= 0.920805 s.e.= 2.348642E-03

A1A2= 1.46204 s.e.= 8.048128E-03

A2A2= 9.277037E-02 s.e.= 7.910053E-04

Mean= 1.07147 s.e.= 4.178579E-03

Mean final relative diversity= 0.994691 s.e.= 2.273077E-05

Mean final diversity reduction= 5.309403E-03 s.e.= 2.273077E-05

Weighted measure of potential recurrent sweep effect= 1.477705E-04

s.e.= 1.178924E-04

**gamma= 1.50000**

Total number of runs= 1023258

Frequency of losses of A2= 0.977271

Mean time to loss = 8.806212E-02 s.e.= 2.716072E-04

Mean weighted relative diversities over paths to loss

A1A1= 0.919733 s.e.= 2.341677E-03

A1A2= 1.46559 s.e.= 8.352458E-03

A2A2= 9.350394E-02 s.e.= 8.194844E-04

Mean= 1.07318 s.e.= 4.304863E-03

Mean final relative diversity= 0.994698 s.e.= 2.327285E-05

Mean final diversity reduction= 5.301654E-03 s.e.= 2.327285E-05

Weighted measure of potential recurrent sweep effect= 2.878068E-04

s.e.= 1.310877E-04

**gamma= 2.00000**

Total number of runs= 1029325

Frequency of losses of A2= 0.971510

Mean time to loss= 8.597763E-02 s.e.= 2.620948E-04

Mean weighted relative diversities over paths to loss

A1A1= 0.923348 s.e.= 2.328638E-03

A1A2= 1.44515 s.e.= 8.036219E-03

A2A2= 9.060447E-02 s.e.= 8.023054E-04

Mean= 1.06883 s.e.= 4.184994E-03

Mean final relative diversity= 0.994936 s.e.= 2.147092E-05

Mean final diversity reduction= 5.064428E-03 s.e.= 2.147092E-05

Weighted measure of potential recurrent sweep effect= -1.183720E-05

s.e.= 1.179314E-04

**gamma= 2.50000**

Total number of runs= 1036671

Frequency of losses of A2= 0.964626

Mean time to loss= 8.358949E-02 s.e.= 2.522966E-04

Mean weighted relative diversities over paths to loss

A1A1= 0.926539 s.e.= 2.312103E-03

A1A2= 1.42519 s.e.= 7.826719E-03

A2A2= 8.786633E-02 s.e.= 7.939737E-04

Mean= 1.06431 s.e.= 4.097005E-03

Mean final relative diversity= 0.995179 s.e.= 2.053961E-05

Mean final diversity reduction= 4.820526E-03 s.e.= 2.053961E-05

Weighted measure of potential recurrent sweep effect= -3.063722E-04

s.e.= 1.089415E-04

**gamma= 3.00000**

Total number of runs= 1044305

Frequency of losses of A2= 0.957575

Mean time to loss= 7.984523E-02 s.e.= 2.354559E-04

Mean weighted relative diversities over paths to loss

A1A1= 0.933542 s.e.= 2.286571E-03

A1A2= 1.38969 s.e.= 7.438420E-03

A2A2= 8.208686E-02 s.e.= 7.609634E-04

Mean= 1.05627 s.e.= 3.924799E-03

Mean final relative diversity= 0.995619 s.e.= 1.778694E-05

Mean final diversity reduction= 4.381061E-03 s.e.= 1.778694E-05

Weighted measure of potential recurrent sweep effect= -7.494616E-04

s.e.= 9.469023E-05

**gamma= 3.50000**

Total number of runs= 1052788

Frequency of losses of A2= 0.949859

Mean time to loss= 7.600689E-02 s.e.= 2.161793E-04

Mean weighted relative diversities over paths to loss

A1A1= 0.941504 s.e.= 2.243865E-03

A1A2= 1.34797 s.e.= 6.863863E-03

A2A2= 7.558378E-02 s.e.= 7.152852E-04

Mean= 1.04671 s.e.= 3.687042E-03

Mean final relative diversity= 0.996049 s.e.= 1.456023E-05

Mean final diversity reduction= 3.951192E-03 s.e.= 1.456023E-05

Weighted measure of potential recurrent sweep effect= -1.256981E-03

s.e.= 7.984043E-05

**gamma= 4.00000**

Total number of runs= 1061113

Frequency of losses of A2= 0.942407

Mean time to loss= 7.214195E-02 s.e.= 1.968146E-04

Mean weighted relative diversities over paths to loss

A1A1= 0.949298 s.e.= 2.197471E-03

A1A2= 1.30709 s.e.= 6.239261E-03

A2A2= 6.892104E-02 s.e.= 6.513679E-03

Mean= 1.03736 s.e.= 3.435434E-03

Mean final relative diversity= 0.996475 s.e.= 1.016121E-05

Mean final diversity reduction= 3.525198E-03 s.e.= 1.016121E-05

Weighted measure of potential recurrent sweep effect= -1.685925E-03

s.e.= 6.706215E-05

**gamma= 4.50000**

Total number of runs= 1070366

Frequency of losses of A2= 0.934260

Mean time to loss= 6.865459E-02 s.e.= 1.792204E-04

Mean weighted relative diversities over paths to loss

A1A1= 0.956998 s.e.= 2.143222E-03

A1A2= 1.27082 s.e.= 5.683585E-03

A2A2= 6.230661E-02 s.e.= 5.964143E-04

Mean= 1.02997 s.e.= 3.199732E-03

Mean final relative diversity= 0.996908 s.e.= NaN

Mean final diversity reduction= 3.091514E-03 s.e.= NaN

Weighted measure of potential recurrent sweep effect= -1.895384E-03

s.e.= 5.406090E-05

**gamma= 5.00000**

Total number of runs= 1079737

Frequency of losses of A2= 0.926151

Mean time to loss= 6.535354E-02 s.e.= 1.621730E-04

Mean weighted relative diversities over paths to loss

A1A1= 0.964127 s.e.= 2.079497E-03

A1A2= 1.23639 s.e.= 5.162108E-03

A2A2= 5.629713E-02 s.e.= 5.466862E-04

Mean= 1.02322 s.e.= 2.973067E-03

Mean final relative diversity= 0.997284 s.e.= NaN

Mean final diversity reduction= 2.715945E-03 s.e.= NaN

Weighted measure of potential recurrent sweep effect= -2.058291E-03

s.e.= 4.629310E-05
