# Supplementary material for: The effects of weak selection on neutral diversity at linked sites
Source: Genetics. 2022 Feb 12;221(1):iyac027. doi: 10.1093/genetics/iyac027 (PMC9071562; doi:10.1093/genetics/iyac027)
Supplement: iyac027_Supplementary_Data [file iyac027_supplementary_data.zip › Supplemental_Table_6_GENETICS-2022-305040.docx]

**Table S6. Losses of deleterious mutations with *h* = 0.1 and no recombination**

**(times are in units of 2*N* generations; diversities are relative to the equilibrium value with no selection)**

**Population size= 50**

**Number of replicate fixations= 1000000**

**Initial A2 allele frequency= 1.00000005E-02**

**gamma= 0.000000**

Total number of runs= 1009835

Frequency of losses of A2= 0.990261

Mean time to loss= 8.636349E-02 s.e.= 2.551485E-04

Mean weighted relative diversities over paths to loss

A1A1= 0.928432 s.e.= 2.317789E-03

A1A2= 1.42281 s.e.= 7.192278E-03

A2A2= 8.707660E-02 s.e.= 7.212389E-04

Mean= 1.06022 s.e.= 3.817672E-03

Mean final relative diversity= 0.994952 s.e.= 1.999532E-05

Mean final diversity reduction= 5.048037E-03 s.e.= 1.999532E-05

Weighted measure of potential recurrent sweep effect= -7.163917E-04

s.e.= 8.776401E-05

**gamma= -0.500000**

Total number of runs= 1008151

Frequency of losses of A2= 0.991915

Mean time to loss (2N generation units)= 8.835453E-02 s.e.= 2.662771E-04

Mean weighted relative diversities over paths to loss

A1A1= 0.923375 s.e.= 2.331458E-03

A1A2= 1.44815 s.e.= 7.559828E-03

A2A2= 9.077421E-02 s.e.= 7.599769E-04

Mean= 1.06644 s.e.= 3.968257E-03

Mean final relative diversity= 0.994762 s.e.= 2.114977E-05

Mean final diversity reduction= 5.238473E-03 s.e.= 2.114977E-05

Weighted measure of potential recurrent sweep effect= -2.368789E-04

s.e.= 9.804322E-05

**gamma= -1.00000**

Total number of runs= 1006550

Frequency of losses of A2= 0.993493

Mean time to loss= 9.021351E-02 s.e.= 2.758472E-04

Mean weighted relative diversities over paths to loss

A1A1= 0.919136 s.e.= 2.342187E-03

A1A2= 1.46960 s.e.= 7.863090E-03

A2A2= 9.397798E-02 s.e.= 7.868985E-04

Mean= 1.07157 s.e.= 4.087179E-03

Mean final relative diversity= 0.994576 s.e.= 2.233412E-05

Mean final diversity reduction= 5.424142E-03 s.e.= 2.233412E-05

Weighted measure of potential recurrent sweep effect= 1.599296E-04

s.e.= 1.081436E-04

**gamma= -1.50000**

Total number of runs= 1005218

Frequency of losses of A2= 0.994809

Mean time to loss (2N generation units)= 9.108339E-02 s.e.= 2.797937E-04

Mean weighted relative diversities over paths to loss

A1A1= 0.917693 s.e.= 2.342735E-03

A1A2= 1.47806 s.e.= 8.057049E-03

A2A2= 9.519815E-02 s.e.= 8.017377E-04

Mean= 1.07378 s.e.= 4.161529E-03

Mean final relative diversity= 0.994478 s.e.= 2.295785E-05

Mean final diversity reduction= 5.522132E-03 s.e.= 2.295785E-05

Weighted measure of potential recurrent sweep effect= 3.250216E-04

s.e.= 1.174222E-04

**gamma= -2.00000**

Total number of runs= 1003922

Frequency of losses of A2= 0.996093

Mean time to loss= 9.177925E-02 s.e.= 2.837412E-04

Mean weighted relative diversities over paths to loss

A1A1= 0.916362 s.e.= 2.352086E-03

A1A2= 1.48726 s.e.= 8.146049E-03

A2A2= 9.617174E-02 s.e.= 8.056773E-04

Mean= 1.07616 s.e.= 4.194904E-03

Mean final relative diversity= 0.994413 s.e.= 2.361145E-05

Mean final diversity reduction= 5.587041E-03 s.e.= 2.361145E-05

Weighted measure of potential recurrent sweep effect= 5.343583E-04

s.e.= 1.181752E-04

**gamma= -2.50000**

Total number of runs= 1002912

Frequency of losses of A2= 0.997096

Mean time to loss= 9.178110E-02 s.e.= 2.811767E-04

Mean weighted relative diversities over paths to loss

A1A1= 0.917987 s.e.= 2.349189E-03

A1A2= 1.47938 s.e.= 7.885681E-03

A2A2= 9.509256E-02 s.e.= 7.801136E-04

Mean= 1.07362 s.e.= 4.092940E-03

Mean final relative diversity= 0.994406 s.e.= 2.337334E-05

Mean final diversity reduction= 5.594194E-03 s.e.= 2.337334E-05

Weighted measure of potential recurrent sweep effect= 2.927821E-04

s.e.= 1.092871E-04

**gamma=-3.00000**

Total number of runs= 1002116

Frequency of losses of A2= 0.997888

Mean time to loss (2N generation units)= 9.170334E-02 s.e.= 2.785753E-04

Mean weighted relative diversities over paths to loss

A1A1= 0.919921 s.e.= 2.346115E-03

A1A2= 1.47175 s.e.= 7.698768E-03

A2A2= 9.382587E-02 s.e.= 7.597607E-04

Mean= 1.07167 s.e.= 4.020164E-03

Mean final relative diversity= 0.994430 s.e.= 2.280763E-05

Mean final diversity reduction= 5.569816E-03 s.e.= 2.280763E-05

Weighted measure of potential recurrent sweep effect= 1.307390E-04

s.e.= 1.039187E-04

**gamma= -3.50000000**

Total number of runs= 1001713

Frequency of losses of A2= 0.998289943

Mean time to loss= 8.98295715E-02 s.e.= 2.68274132E-04

Predicted approximate mean time to loss= 9.35306475E-02

Mean weighted relative diversities over paths to loss

A1A1= 0.924704254 s.e.= 2.34394963E-03

A1A2= 1.44823444 s.e.= 7.24801887E-03

A2A2= 9.00679380E-02 s.e.= 7.15371105E-04

Mean= 1.06576860 s.e.= 3.85630853E-03

Mean final relative diversity= 0.994592130 s.e.= 2.21260707E-05

Mean final diversity reduction= 5.40786982E-03 s.e.= 2.21260707E-05

Weighted measure of potential recurrent sweep effect= -3.73616407E-04

s.e.= 8.93040778E-05

**Gamma= -4.00000**

Total number of runs= 1001089

Frequency of losses of A2= 0.998912

Mean time to loss= 8.960006E-02 s.e.= 2.617796E-04

Mean weighted relative diversities over paths to loss

A1A1= 0.929416 s.e.= 2.328044E-03

A1A2= 1.42997 s.e.= 6.943138E-03

A2A2= 8.701151E-02 s.e.= 6.736401E-04

Mean= 1.06044 s.e.= 3.718333E-03

Mean final relative diversity= 0.994684 s.e.= 2.032549E-05

Mean final diversity reduction= 5.316317E-03 s.e.= 2.032549E-05

Weighted measure of potential recurrent sweep effect= -7.731948E-04

s.e.= 8.374372E-05

**gamma= -4.50000**

Total number of runs= 1000798

Frequency of losses of A2= 0.999203

Mean time to loss= 8.835666E-02 s.e.= 2.530824E-04

Mean weighted relative diversities over paths to loss

A1A1= 0.934292 s.e.= 2.318533E-03

A1A2= 1.40939 s.e.= 6.561878E-03

A2A2= 8.341775E-02 s.e.= 6.289684E-04

Mean= 1.05511 s.e.= 3.566838E-03

Mean final relative diversity= 0.994848 s.e.= 1.883150E-05

Mean final diversity reduction= 5.151510E-03 s.e.= 1.883150E-05

Weighted measure of potential recurrent sweep effect= -1.153610E-03

s.e.= 7.253952E-05

**gamma= -5.00000**

Total number of runs= 1000532

Frequency of losses of A2= 0.999468

Mean time to loss= 8.708624E-02 s.e.= 2.448787E-04

Mean weighted relative diversities over paths to loss

A1A1= 0.938890 s.e.= 2.306646E-03

A1A2= 1.39057 s.e.= 6.284140E-03

A2A2= 8.004992E-02 s.e.= 5.932208E-04

Mean= 1.05068 s.e.= 3.453440E-03

Mean final relative diversity= 0.995025 s.e.= 1.695768E-05

Mean final diversity reduction= 4.975498E-03 s.e.= 1.695768E-05

Weighted measure of potential recurrent sweep effect= -1.433697E-03

s.e.= 6.610242E-05
