# Supplementary material for: The effects of weak selection on neutral diversity at linked sites
Source: Genetics. 2022 Feb 12;221(1):iyac027. doi: 10.1093/genetics/iyac027 (PMC9071562; doi:10.1093/genetics/iyac027)
Supplement: iyac027_Supplementary_Data [file iyac027_supplementary_data.zip › Supplemental_Table_7_GENETICS-2022-305040.docx]

**Table S7. Losses of favorable mutations with *h* = 0.9 and no recombination**

**(times are in units of 2*N* generations; diversities are relative to the equilibrium value with no selection)**

**Population size= 500**

**Number of replicate losses= 1000000**

**Initial A2 allele frequency= 1.00000005E-03**

**gamma= 0.500000000**

Total number of runs= 1001363

Frequency of losses of A2= 0.998638868

Mean time to loss= 1.32749947E-02 s.e.= 9.08038564E-05

Mean weighted relative diversities over paths to loss

A1A1= 0.947605491 s.e.= 5.37927030E-03

A1A2= 1.31877780 s.e.= 1.70496777E-02

A2A2= 6.01948351E-02 s.e.= 1.65475463E-03

Mean= 1.04745078 s.e.= 8.96127243E-03

Mean final relative diversity= 0.999499500 s.e.= 6.93722222E-06

Mean final diversity reduction= 5.00500202E-04 s.e.= 6.93722222E-06

Weighted measure of potential recurrent sweep effect= 3.35514378E-05

s.e.= 3.33994103E-05

**gamma= 1.00000000**

Total number of runs= 1001813

Frequency of losses of A2= 0.998190284

Mean time to loss= 1.34064117E-02 s.e.= 9.07118811E-05

Mean weighted relative diversities over paths to loss

A1A1= 0.947206140 s.e.= 5.32099511E-03

A1A2= 1.31528687 s.e.= 1.65789369E-02

A2A2= 6.07830733E-02 s.e.= 1.65933208E-03

Mean= 1.04696763 s.e.= 8.82992242E-03

Mean final relative diversity= 0.999485672 s.e.= 7.01783802E-06

Mean final diversity reduction= 5.14328480E-04 s.e.= 7.01783802E-06

Weighted measure of potential recurrent sweep effect= 1.60047475E-05

s.e.= 3.27998387E-05

**gamma= 1.50000000**

Total number of runs= 1002324

Frequency of losses of A2= 0.997681379

Mean time to loss= 1.33340470E-02 s.e.= 9.32070616E-0

Mean weighted relative diversities over paths to loss

A1A1= 0.944327056 s.e.= 5.38034644E-03

A1A2= 1.33468831 s.e.= 1.84714701E-02

A2A2= 6.27329051E-02 s.e.= 1.80047506E-03

Mean= 1.05332375 s.e.= 9.59986169E-03

Mean final relative diversity= 0.999490440 s.e.= 6.86021485E-06

Mean final diversity reduction= 5.09560108E-04 s.e.= 6.86021485E-06

Weighted measure of potential recurrent sweep effect= 1.02037731E-04

s.e.= 4.17922929E-05

**gamma= 2.00000000**

Total number of runs= 1002970

Frequency of losses of A2= 0.997038782

Mean time to loss= 1.30731976E-02 s.e.= 8.78753490E-05

Mean weighted relative diversities over paths to loss

A1A1= 0.950090945 s.e.= 5.29478164E-03

A1A2= 1.30223572 s.e.= 1.69618241E-02

A2A2= 5.80647178E-02 s.e.= 1.62341027E-03

Mean= 1.04488754 s.e.= 8.87313951E-03

Mean final relative diversity= 0.999521375 s.e.= 6.07762604E-06

Mean final diversity reduction= 4.78625298E-04 s.e.= 6.07762604E-06

Weighted measure of potential recurrent sweep effect= 1.06165271E-05

s.e.= 3.48601970E-05

**gamma= 2.50000000**

Total number of runs= 1003680

Frequency of losses of A2= 0.996333480

Mean time to loss (2N generation units)= 1.27950525E-02 s.e.= 8.39413333E-05

Predicted approximate mean time to loss= 1.12914424E-02

Mean weighted relative diversities over paths to loss

A1A1= 0.952192485 s.e.= 5.17589273E-03

A1A2= 1.28145790 s.e.= 1.58907790E-02

A2A2= 5.61693981E-02 s.e.= 1.61506340E-03

Mean= 1.04066968 s.e.= 8.49661324E-03

Mean final relative diversity= 0.999538898 s.e.= 5.98957740E-06

Mean final diversity reduction= 4.61101532E-04 s.e.= 5.98957740E-06

Weighted measure of potential recurrent sweep effect= -3.91868743E-05

s.e.= 2.96634280E-05

**gamma= 3.00000000**

Total number of runs= 1004379

Frequency of losses of A2= 0.995640099

Mean time to loss= 1.27116470E-02 s.e.= 8.07836259E-05

Mean weighted relative diversities over paths to loss

A1A1= 0.955130935 s.e.= 5.10752061E-03

A1A2= 1.26275229 s.e.= 1.42433448E-02

A2A2= 5.37467040E-02 s.e.= 1.48891122E-03

Mean= 1.03485906 s.e.= 7.85566401E-03

Mean final relative diversity= 0.999543011 s.e.= 6.52399740E-06

Mean final diversity reduction= 4.56988811E-04 s.e.= 6.52399740E-06

Weighted measure of potential recurrent sweep effect= -1.08948618E-04

s.e.= 2.25595159E-05

**gamma= 3.50000000**

Total number of runs= 1005245

Frequency of losses of A2= 0.994782388

Mean time to loss= 1.21613536E-02 s.e.= 7.60999756E-05

Mean weighted relative diversities over paths to loss

A1A1= 0.959888101 s.e.= 4.98246774E-03

A1A2= 1.24383485 s.e.= 1.53954187E-02

A2A2= 4.89755236E-02 s.e.= 1.54010602E-03

Mean= 1.03343880 s.e.= 8.20278563E-03

Mean final relative diversity= 0.999607027 s.e.= 4.87980788E-06

Mean final diversity reduction= 3.92973423E-04 s.e.= 4.87980788E-06

Weighted measure of potential recurrent sweep effect= -8.21669091E-05

s.e.= 3.05057038E-05

**gamma= 4.00000000**

Total number of runs= 1006088

Frequency of losses of A2= 0.993948817

Mean time to loss= 1.16906054E-02 s.e.= 6.87672073E-05

Mean weighted relative diversities over paths to loss

A1A1= 0.965499878 s.e.= 4.81655216E-03

A1A2= 1.20770359 s.e.= 1.24497414E-02

A2A2= 4.35612164E-02 s.e.= 1.32378784E-03

Mean= 1.02424812 s.e.= 7.00530456E-03

Mean final relative diversity= 0.999662340 s.e.= 2.35849643E-06

Mean final diversity reduction= 3.37660313E-04 s.e.= 2.35849643E-06

Weighted measure of potential recurrent sweep effect= -1.51665387E-04

s.e.= 1.56681854E-05

**gamma= 4.50000000**

Total number of runs= 1007008

Frequency of losses of A2= 0.993040740

Mean time to loss= 1.12785390E-02 s.e.= 6.30418799E-05

Mean weighted relative diversities over paths to loss

A1A1= 0.971570313 s.e.= 4.70391288E-03

A1A2= 1.18139088 s.e.= 1.13333752E-02

A2A2= 3.87074947E-02 s.e.= 1.17973250E-03

Mean= 1.01947105 s.e.= 6.56867493E-03

Mean final relative diversity= 0.999691725 s.e.= 2.13600197E-06

Mean final diversity reduction= 3.08275223E-04 s.e.= 2.13600197E-06

Weighted measure of potential recurrent sweep effect= -1.80941410E-04

s.e.= 1.45767872E-05

**gamma= 5.00000000**

Total number of runs= 1007849

Frequency of losses of A2= 0.992212117

Mean time to loss= 1.10617662E-02 s.e.= 5.93508412E-05

Predicted approximate mean time to loss= 4.03364003E-03

Mean weighted relative diversities over paths to loss

A1A1= 0.975361884 s.e.= 4.58973972E-03

A1A2= 1.16428852 s.e.= 1.04451999E-02

A2A2= 3.50561552E-02 s.e.= 1.04892300E-03

Mean= 1.01625144 s.e.= 6.16741134E-03

Mean final relative diversity= 0.999724329 s.e.= NaN

Mean final diversity reduction= 2.75671482E-04 s.e.= NaN

Weighted measure of potential recurrent sweep effect= -1.90489780E-04

s.e.= 1.17814934E-05
