# Supplementary material for: The effects of weak selection on neutral diversity at linked sites
Source: Genetics. 2022 Feb 12;221(1):iyac027. doi: 10.1093/genetics/iyac027 (PMC9071562; doi:10.1093/genetics/iyac027)
Supplement: iyac027_Supplementary_Data [file iyac027_supplementary_data.zip › Supplemental_Table_8_GENETICS-2022-305040.docx]

**Table S8. Losses of deleterious mutations with *h* = 0.1 and no recombination**

**(times are in units of 2*N* generations; diversities are relative to the equilibrium value with no selection)**

**Population size= 500**

**Number of replicate losses= 1000000**

**Initial A2 allele frequency= 1.00000005E-03**

**gamma= 0.00000000**

Total number of runs= 1001006

Frequency of losses of A2= 0.998995006

Mean time to loss (2N generation units)= 1.30741727E-02 s.e.= 8.73775716E-05

Predicted approximate mean time to loss= 1.29699102E-02

Mean weighted relative diversities over paths to loss

A1A1= 0.950678706 s.e.= 5.28616970E-03

A1A2= 1.29879248 s.e.= 1.62449311E-02

A2A2= 5.76196611E-02 s.e.= 1.61997566E-03

Mean= 1.04362953 s.e.= 8.69332813E-03

Mean final relative diversity= 0.999521434 s.e.= 6.03635135E-06

Mean final diversity reduction= 4.78565693E-04 s.e.= 6.03635135E-06

Weighted measure of potential recurrent sweep effect= -5.99234500E-06

s.e.= 3.15845245E-05

**gamma= -0.500000000**

Total number of runs= 1000804

Frequency of losses of A2= 0.999196649

Mean time to loss= 1.32603729E-02 s.e.= 8.83028406E-05

Mean weighted relative diversities over paths to loss

A1A1= 0.950881004 s.e.= 5.33350045E-03

A1A2= 1.30161095 s.e.= 1.57002453E-02

A2A2= 5.80196008E-02 s.e.= 1.52333407E-03

Mean= 1.04327083 s.e.= 8.47336929E-03

Mean final relative diversity= 0.999498665 s.e.= 6.65676680E-06

Mean final diversity reduction= 5.01334667E-04 s.e.= 6.65676680E-06

Weighted measure of potential recurrent sweep effect= -2.54480765E-05

s.e.= 2.82750516E-05

**gamma= -1.00000000**

Total number of runs= 1000645

Frequency of losses of A2= 0.999355435

Mean time to loss= 1.35396691E-02 s.e.= 9.29076195E-05

Mean weighted relative diversities over paths to loss

A1A1= 0.945555091 s.e.= 5.40263252E-03

A1A2= 1.32822824 s.e.= 1.66183263E-02

A2A2= 6.19939603E-02 s.e.= 1.64474850E-03

Mean= 1.04922199 s.e.= 8.88812914E-03

Mean final relative diversity= 0.999467194 s.e.= 7.50833260E-06

Mean final diversity reduction= 5.32805920E-04 s.e.= 7.50833260E-06

Weighted measure of potential recurrent sweep effect= 3.69567424E-05

s.e.= 3.14103818E-05

**gamma= -1.50000000**

Total number of runs= 1000553

Frequency of losses of A2= 0.999447286

Mean time to loss = 1.35535272E-02 s.e.= 9.43302657E-05

Predicted approximate mean time to loss= 1.35324234E-02

Mean weighted relative diversities over paths to loss

A1A1= 0.944491863 s.e.= 5.42611070E-03

A1A2= 1.33772969 s.e.= 1.82715934E-02

A2A2= 6.36546686E-02 s.e.= 1.77330431E-03

Mean= 1.05329955 s.e.= 9.54427943E-03

Mean final relative diversity= 0.999464750 s.e.= 7.15891429E-06

Mean final diversity reduction= 5.35249710E-04 s.e.= 7.15891429E-06

Weighted measure of potential recurrent sweep effect= 8.80955267E-05

s.e.= 4.33751593E-05

**gamma= -2.00000000**

Total number of runs= 1000376

Frequency of losses of A2= 0.999624133

Mean time to loss= 1.37915919E-02 s.e.= 9.65006111E-05

Predicted approximate mean time to loss= 1.36736119E-02

Mean weighted relative diversities over paths to loss

A1A1= 0.942242563 s.e.= 5.46363369E-03

A1A2= 1.34828985 s.e.= 1.73986983E-02

A2A2= 6.50543794E-02 s.e.= 1.71170221E-03

Mean= 1.05414033 s.e.= 9.22858249E-03

Mean final relative diversity= 0.999444067 s.e.= 7.77415971E-06

Mean final diversity reduction= 5.55932522E-04 s.e.= 7.77415971E-06

Weighted measure of potential recurrent sweep effect= 9.22322724E-05

s.e.= 3.53088835E-05

**gamma= -2.50000000**

Total number of runs= 1000319

Frequency of losses of A2= 0.999681115

Mean time to loss= 1.37901315E-02 s.e.= 9.55532887E-05

Mean weighted relative diversities over paths to loss

A1A1= 0.944637239 s.e.= 5.43255126E-03

A1A2= 1.34146357 s.e.= 1.77856088E-02

A2A2= 6.33159578E-02 s.e.= 1.68140372E-03

Mean= 1.05320168 s.e.= 9.35363956E-03

Mean final relative diversity= 0.999453425 s.e.= 7.09753795E-06

Mean final diversity reduction= 5.46574593E-04 s.e.= 7.09753795E-06

Weighted measure of potential recurrent sweep effect= 8.61262452E-05

s.e.= 4.02411824E-05

**gamma= -3.00000000**

Total number of runs= 1000212

Frequency of losses of A2= 0.999788046

Mean time to loss (2N generation units)= 1.36317722E-02 s.e.= 9.50253307E-05

Predicted approximate mean time to loss= 1.38865178E-02

Mean weighted relative diversities over paths to loss

A1A1= 0.945501924 s.e.= 5.47824427E-03

A1A2= 1.34145820 s.e.= 1.86509080E-02

A2A2= 6.26780987E-02 s.e.= 1.68695976E-03

Mean= 1.05327928 s.e.= 9.65534057E-03

Mean final relative diversity= 0.999461412 s.e.= 7.13267491E-06

Mean final diversity reduction= 5.38587570E-04 s.e.= 7.13267491E-06

Weighted measure of potential recurrent sweep effect= 8.86946873E-05

s.e.= 4.63490251E-05

**gamma= -3.50000000**

Total number of runs= 1000145

Frequency of losses of A2= 0.999855042

Mean time to loss= 1.35570522E-02 s.e.= 9.11643438E-05

Mean weighted relative diversities over paths to loss

A1A1= 0.949535489 s.e.= 5.42131811E-03

A1A2= 1.31581163 s.e.= 1.58072282E-02

A2A2= 5.95157035E-02 s.e.= 1.48978538E-03

Mean= 1.04536760 s.e.= 8.52853712E-03

Mean final relative diversity= 0.999466598 s.e.= 7.02673833E-06

Mean final diversity reduction= 5.33401966E-04 s.e.= 7.02673833E-06

Weighted measure of potential recurrent sweep effect= -1.65814163E-05 s.e.= 2.82989295E-05

**gamma= -4.00000000**

Total number of runs= 1000119

Frequency of losses of A2= 0.999881029

Mean time to loss= 1.35067469E-02 s.e.= 8.91531381E-05

Mean weighted relative diversities over paths to loss

A1A1= 0.951178849 s.e.= 5.35744801E-03

A1A2= 1.30268240 s.e.= 1.48146637E-02

A2A2= 5.83402738E-02 s.e.= 1.45302061E-03

Mean= 1.04229975 s.e.= 8.17834493E-03

Mean final relative diversity= 0.999474227 s.e.= 6.87841157E-06

Mean final diversity reduction= 5.25772572E-04 s.e.= 6.87841157E-06

Weighted measure of potential recurrent sweep effect= -5.16191394E-05

s.e.= 2.39516648E-05

**gamma= -4.50000000**

Total number of runs= 1000075

Frequency of losses of A2= 0.999925017

Mean time to loss= 1.33820074E-02 s.e.= 8.68187781E-05

Mean weighted relative diversities over paths to loss

A1A1= 0.955757737 s.e.= 5.36965625E-03

A1A2= 1.28932905 s.e.= 1.43804541E-02

A2A2= 5.51173687E-02 s.e.= 1.34662713E-03

Mean= 1.03918445 s.e.= 7.97667634E-03

Mean final relative diversity= 0.999498785 s.e.= 6.02080036E-06

Mean final diversity reduction= 5.01215458E-04 s.e.= 6.02080036E-06

Weighted measure of potential recurrent sweep effect= -7.44553254E-05

s.e.= 2.31675403E-05

**gamma= -5.00000000**

Total number of runs= 1000063

Frequency of losses of A2= 0.999936998

Mean time to loss= 1.31812040E-02 s.e.= 8.19022389E-05

Mean weighted relative diversities over paths to loss

A1A1= 0.960606456 s.e.= 5.23654418E-03

A1A2= 1.26077032 s.e.= 1.30846687E-02

A2A2= 5.10573462E-02 s.e.= 1.21017103E-03

Mean= 1.03225398 s.e.= 7.41160056E-03

Mean final relative diversity= 0.999516368 s.e.= 5.66789504E-06

Mean final diversity reduction= 4.83632088E-04 s.e.= 5.66789504E-06

Weighted measure of potential recurrent sweep effect= -1.54419424E-04

s.e.= 1.87625974E-05
