# Supplementary material for: The effects of weak selection on neutral diversity at linked sites
Source: Genetics. 2022 Feb 12;221(1):iyac027. doi: 10.1093/genetics/iyac027 (PMC9071562; doi:10.1093/genetics/iyac027)
Supplement: iyac027_Supplementary_Data [file iyac027_supplementary_data.zip › Supplemental_Table_9_GENETICS-2022-305040.docx]

**Table S9. Diversity statistics for fixations and losses of favorable and deleterious**

**mutations with different dominance coefficients. The table display the means and**

**standard errors of statistics obtained from the simulations described in the text. *π_w_***

**is the mean relative diversity during the course of a fixation or loss event, Δ*π*_0_ is the mean deviation of the relative diversity from 1 at the end of an event; Δ*π_S_* is calculated from**

**Equation (10); *t** and *t*** are the mean times to fixation and loss, respectively. *N* = 50**.

**Fixations**

| *h* | *π_w_* | Δ*π*_0_ | Δ*π_S_* | *t** |
| --- | --- | --- | --- | --- |
| 0 | 1.1679±0.0029 | –0.4351±0.0003 | –0.1261±0.0026 | 1.8407±0.0030 |
| 0.1 | 1.1726±0.0030 | –0.4341±0.0003 | –0.1056±0.0028 | 1.8633±0.0031 |
| 0.2 | 1.1791±0.0030 | –0.4324±0.0003 | –0.0963±0.0028 | 1.8784±0.0031 |
| 0.3 | 1.1858±0.0030 | –0.4305±0.0003 | –0.0772±0.0029 | 1.9001±0.0032 |
| 0.4 | 1.1921±0.0031 | –0.4297±0.0003 | –0.0610±0.0030 | 1.9198±0.0032 |
| 0.5 | 1.1997±0.0032 | –0.4291±0.0003 | –0.0420±0.0031 | 1.9384±0.0032 |
| 0.6 | 1.2055±0.0032 | –0.4280±0.0003 | –0.0256±0.0032 | 1.9565±0.0033 |
| 0.7 | 1.2143±0.0033 | –0.4263±0.0003 | –0.0013±0.0034 | 1.9830±0.0034 |
| 0.8 | 1.2177±0.0033 | –0.4259±0.0003 | 0.00841±0.0034 | 1.9953±0.0035 |
| 0.9 | 1.2244±0.0033 | –0.4243±0.0003 | 0.0291±0.0035 | 2.0199±0.0035 |
| 1.0 | 1.2357±0.0034 | –0.4225±0.0003 | 0.06075±0.0037 | 2.0504±0.0036 |

*γ* = 1.0

*γ* = 1.5

| 0 | 1.1450±0.0027 | –0.4407±0.0003 | –0.1846±0.0023 | 1.7665±0.0028 |
| --- | --- | --- | --- | --- |
| 0.1 | 1.1524±0.0028 | –0.4392±0.0003 | –0.1661±0.0024 | 1.7917±0.0030 |
| 0.2 | 1.1610±0.0029 | –0.4371±0.0003 | –0.1446±0.0025 | 1.8172±0.0030 |
| 0.3 | 1.1714±0.0029 | –0.4350±0.0003 | –0.1178±0.0027 | 1.8502±0.0031 |
| 0.4 | 1.1798±0.0030 | –0.4320±0.0003 | –0.0940±0.0028 | 1.8795±0.0031 |
| 0.5 | 1.1876±0.0031 | –0.4310±0.0003 | –0.0743±0.0029 | 1.9017±0.0032 |
| 0.6 | 1.1980±0.0032 | –0.4290±0.0003 | –0.0459±0.0031 | 1.9351±0.0033 |
| 0.7 | 1.2072±0.0032 | –0.4273±0.0003 | –0.0204±0.0032 | 1.9632±0.0033 |
| 0.8 | 1.2217±0.0033 | –0.4249±0.0003 | 0.00850±0.0034 | 1.9950±0.0035 |
| 0.9 | 1.2301±0.0034 | –0.4231±0.0003 | 0.0448±0.00356 | 2.0336±0.0036 |
| 1.0 | 1.2409±0.0035 | –0.4216±0.0003 | 0.0755±0.0038 | 2.0637±0.0037 |

*γ* = –1.0

| *h* | *π_w_* | Δ*π*_0_ | Δ*π_S_* | *t** |
| --- | --- | --- | --- | --- |
| 0 | 1.2343±0.0034 | –0.4252±0.0003 | 0.0530±0.0037 | 2.0407±0.0036 |
| 0.1 | 1.2283±0.0033 | –0.4250±0.0003 | 0.0381±0.0035 | 2.0280±0.0035 |
| 0.2 | 1.2204±0.0033 | –0.4260±0.0003 | 0.0161±0.0034 | 2.0052±0.0035 |
| 0.3 | 1.2151±0.0033 | –0.4270±0.0003 | –0.0002±0.0034 | 1.9839±0.0034 |
| 0.4 | 1.2047±0.0032 | –0.4278±0.0003 | –0.0271±0.0032 | 1.9569±0.0034 |
| 0.5 | 1.2008±0.0032 | –0.4295±0.0003 | –0.0401±0.0031 | 1.9380±0.0033 |
| 0.6 | 1.1939±0.0031 | –0.4292±0.0003 | –0.0559±0.0030 | 1.9253±0.0033 |
| 0.7 | 1.1879±0.0031 | –0.4300±0.0003 | –0.0716±0.0030 | 1.9091±0.0032 |
| 0.8 | 1.1794±0.0030 | –0.4314±0.0003 | –0.0943±0.0028 | 1.8796±0.0031 |
| 0.9 | 1.1739±0.0030 | –0.4328±0.0003 | –0.1091±0.0027 | 1.8611±0.0031 |
| 1.0 | 1.1691±0.0029 | –0.4334±0.0003 | –0.1214±0.0027 | 1.8453±0.0031 |

*γ* = –1.5

| 0 | 1.2406±0.0034 | –0.4246±0.0003 | 0.0712±0.0038 | 2.0611±0.0036 |
| --- | --- | --- | --- | --- |
| 0.1 | 1.2333±0.0034 | –0.4249±0.0003 | 0.0484±0.0036 | 2.0380±0.0036 |
| 0.2 | 1.2221±0.0034 | –0.4276±0.0003 | 0.0176±0.0035 | 2.0041±0.0035 |
| 0.3 | 1.2088±0.0034 | –0.4273±0.0003 | –0.0158±0.0032 | 1.9709±0.0034 |
| 0.4 | 1.1994±0.0032 | –0.4295±0.0003 | –0.0435±0.0031 | 1.9361±0.0033 |
| 0.5 | 1.1885±0.0031 | –0.4304±0.0003 | –0.0710±0.0029 | 1.9070±0.0032 |
| 0.6 | 1.1776±0.0030 | –0.4327±0.0003 | –0.1004±0.0028 | 1.8711±0.0031 |
| 0.7 | 1.1696±0.0029 | –0.4331±0.0003 | –0.1196±0.0027 | 1.8484±0.0031 |
| 0.8 | 1.1612±0.0029 | –0.4344±0.0003 | –0.1397±0.0026 | 1.8230±0.0030 |
| 0.9 | 1.1545±0.0029 | –0.4356±0.0003 | –0.1576±0.0025 | 1.8002±0.0030 |
| 1.0 | 1.1448±0.0028 | –0.4373±0.0003 | –0.1807±0.0023 | 1.7716±0.0029 |

**Losses**

| *h* | *π_w_* | Δ*π*_0_ | Δ*π_S_* | *t*** |
| --- | --- | --- | --- | --- |
| 0 | 1.0507±0.0036 | –0.00445±0.00002 | –0.00120±0.00008 | 0.0815±0.0002 |
| 0.1 | 1.0503±0.0036 | –0.00450±0.00002 | –0.00126±0.00007 | 0.0819±0.0002 |
| 0.2 | 1.0544±0.0037 | –0.00460±0.00002 | –0.00097±0.00008 | 0.0829±0.0002 |
| 0.3 | 1.0560±0.0037 | –0.00469±0.00002 | –0.00091±0.00008 | 0.0834±0.0002 |
| 0.4 | 1.0559±0.0037 | –0.00477±0.00002 | –0.00095±0.00008 | 0.0839±0.0002 |
| 0.5 | 1.0608±0.0039 | –0.00492±0.00002 | –0.00061±0.00009 | 0.0851±0.0003 |
| 0.6 | 1.0629±0.0039 | –0.00498±0.00002 | –0.00055±0.00009 | 0.0857±0.0003 |
| 0.7 | 1.0673±0.0040 | –0.00508±0.00002 | –0.00012±0.00010 | 0.0866±0.0003 |
| 0.8 | 1.0710±0.0041 | –0.00521±0.00002 | 0.00014±0.00011 | 0.0879±0.0003 |
| 0.9 | 1.0743±0.0042 | –0.00534±0.00002 | 0.00039±0.00011 | 0.0887±0.0003 |
| 1.0 | 1.0759±0.0042 | –0.00543±0.00002 | 0.00049±0.00011 | 0.0895±0.0003 |

*γ* = 1.0

*γ* = 1.5

| *h* | *π_w_* | Δ*π*_0_ | Δ*π_S_* | *t*** |
| --- | --- | --- | --- | --- |
| 0 | 1.0409±0.0032 | –0.00414±0.00001 | –0.00180±0.00006 | 0.0789±0.0002 |
| 0.1 | 1.0435±0.0034 | –0.00426±0.00001 | –0.00167±0.00006 | 0.0797±0.0002 |
| 0.2 | 1.0473±0.0035 | –0.00442±0.00002 | –0.00146±0.00007 | 0.0810±0.0002 |
| 0.3 | 1.0521±0.0036 | –0.00454±0.00002 | –0.00126±0.00007 | 0.0822±0.0002 |
| 0.4 | 1.0528±0.0037 | –0.00464±0.00002 | –0.00114±0.00008 | 0.0827±0.0002 |
| 0.5 | 1.0555±0.0037 | –0.00475±0.00002 | –0.00097±0.00008 | 0.0839±0.0002 |
| 0.6 | 1.0585±0.0038 | –0.00488±0.00002 | –0.00079±0.00009 | 0.0849±0.0003 |
| 0.7 | 1.0642±0.0040 | –0.00499±0.00002 | –0.00037±0.00010 | 0.0857±0.0003 |
| 0.8 | 1.0695±0.0041 | –0.00512±0.00002 | 0.00006±0.00010 | 0.0871±0.0003 |
| 0.9 | 1.0741±0.0041 | –0.00527±0.00002 | 0.00014±0.00011 | 0.0881±0.0003 |
| 1.0 | 1.0775±0.0043 | –0.00540±0.00002 | 0.00064±0.00012 | 0.0893±0.0003 |

*γ* = –1.0

| 0 | 1.060±0.0014 | –0.00324±0.00001 | 0.00087±0.00004 | 0.0876±0.0001 |
| --- | --- | --- | --- | --- |
| 0.1 | 1.0546±0.0014 | –0.00311±0.00001 | 0.00044±0.00004 | 0.0862±0.0001 |
| 0.2 | 1.0505±0.0014 | –0.00300±0.00001 | 0.00015±0.00003 | 0.0850±0.0001 |
| 0.3 | 1.0467±0.0013 | –0.00292±0.00001 | –0.00012±0.00003 | 0.0840±0.0001 |
| 0.4 | 1.0432±0.0013 | –0.00281±0.00001 | –0.00033±0.00003 | 0.0829±0.0001 |
| 0.5 | 1.0396±0.0013 | –0.00270±0.00001 | –0.00053±0.00003 | 0.0818±0.0001 |
| 0.6 | 1.0355±0.0012 | –0.00261±0.00001 | –0.00079±0.00003 | 0.0807±0.0001 |
| 0.7 | 1.0319±0.0012 | –0.00251±0.00001 | –0.00099±0.00002 | 0.0796±0.0001 |
| 0.8 | 1.0294±0.0012 | –0.00242±0.00001 | –0.00111±0.00002 | 0.0788±0.0001 |
| 0.9 | 1.0263±0.0011 | –0.00233±0.00001 | –0.00126±0.00002 | 0.0777±0.0001 |
| 1.0 | 1.0235±0.0012 | –0.00225±0.00001 | –0.00140±0.00002 | 0.0769±0.0001 |

*γ* = –1.5

| 0 | 1.0846±0.0044 | –0.00586±0.00003 | 0.00119±0.00013 | 0.0935±0.0003 |
| --- | --- | --- | --- | --- |
| 0.1 | 1.0780±0.0042 | –0.00555±0.00002 | 0.000651±0.00012 | 0.0908±0.0003 |
| 0.2 | 1.0728±0.0041 | –0.00535±0.00002 | 0.00026±0.00011 | 0.0891±0.0003 |
| 0.3 | 1.0667±0.0040 | –0.00514±0.00002 | –0.00019±0.00010 | 0.0873±0.0003 |
| 0.4 | 1.0619±0.0039 | –0.00495±0.00002 | –0.00053±0.00009 | 0.0855±0.0003 |
| 0.5 | 1.0583±0.0038 | –0.00479±0.00002 | –0.00076±0.00008 | 0.0842±0.0002 |
| 0.6 | 1.0551±0.0036 | –0.00455±0.00002 | –0.00123±0.00008 | 0.0819±0.0002 |
| 0.7 | 1.0493±0.0036 | –0.00439±0.00002 | –0.00128±0.00008 | 0.0807±0.0002 |
| 0.8 | 1.0455±0.0035 | –0.00427±0.00002 | –0.00152±0.00007 | 0.0795±0.0002 |
| 0.9 | 1.0433±0.0034 | –0.00407±0.00001 | –0.00157±0.00006 | 0.0780±0.0002 |
| 1.0 | 1.0038±0.0033 | –0.00392±0.00001 | –0.00190±0.00006 | 0.0765±0.0002 |
