# Supplementary figures and images for: The effects of weak selection on neutral diversity at linked sites
Source: Genetics. 2022 Feb 12;221(1):iyac027. doi: 10.1093/genetics/iyac027 (PMC9071562; doi:10.1093/genetics/iyac027)

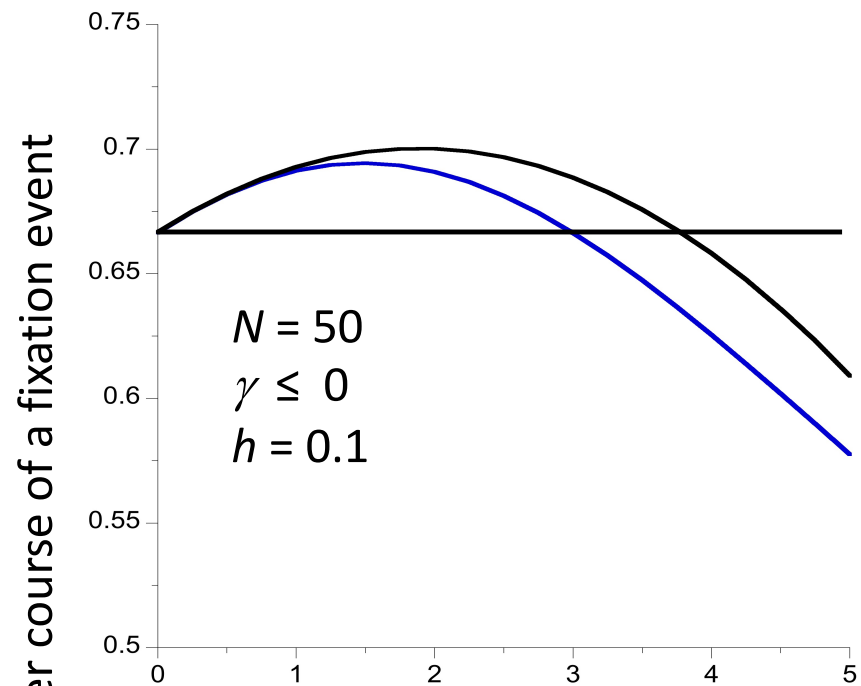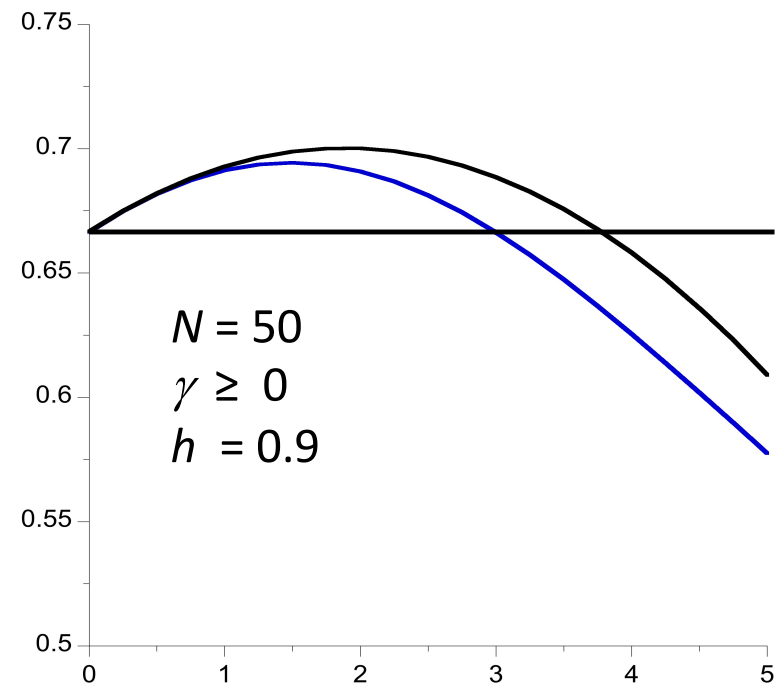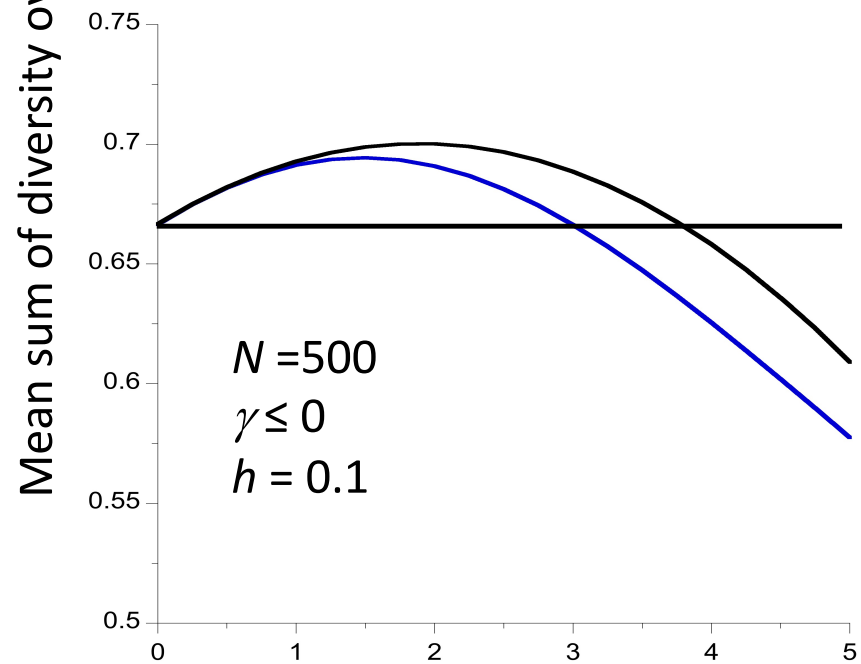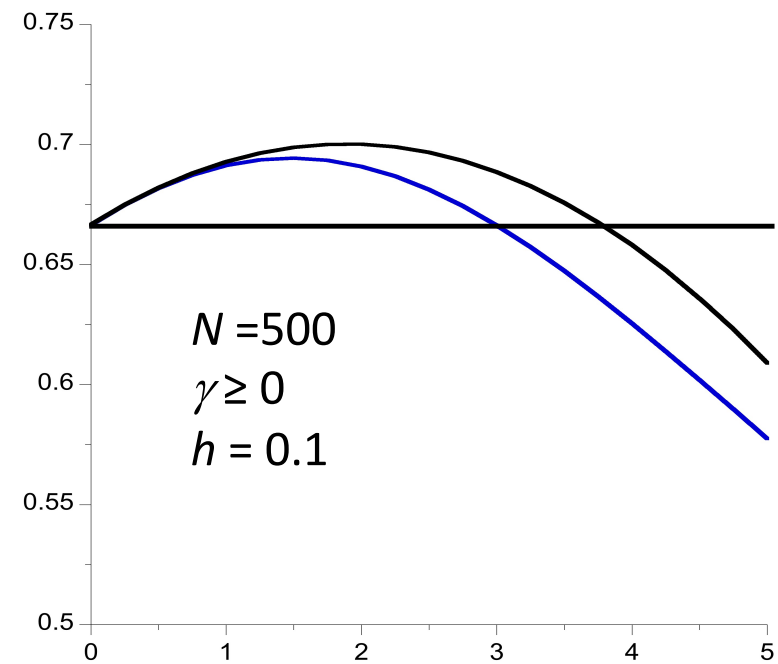

Absolute value of  $\gamma = 2N_e s$

Supplement: iyac027_Supplementary_Data [file iyac027_supplementary_data.zip › Supplemental_Figure_1_GENETICS-2022-305040.pdf]

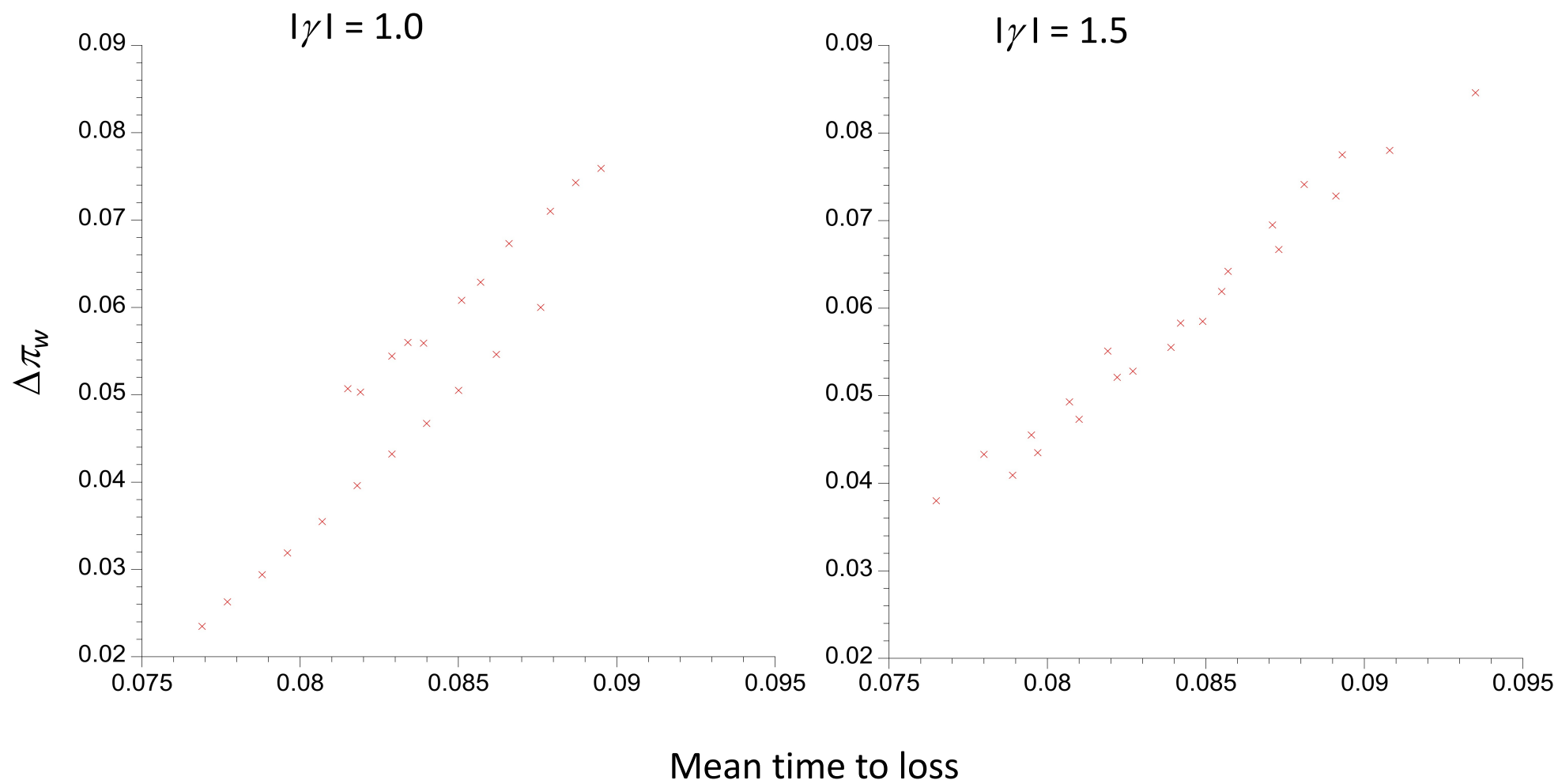

Supplement: iyac027_Supplementary_Data [file iyac027_supplementary_data.zip › Supplemental_Figure_10_GENETICS-2022-305040.pdf]

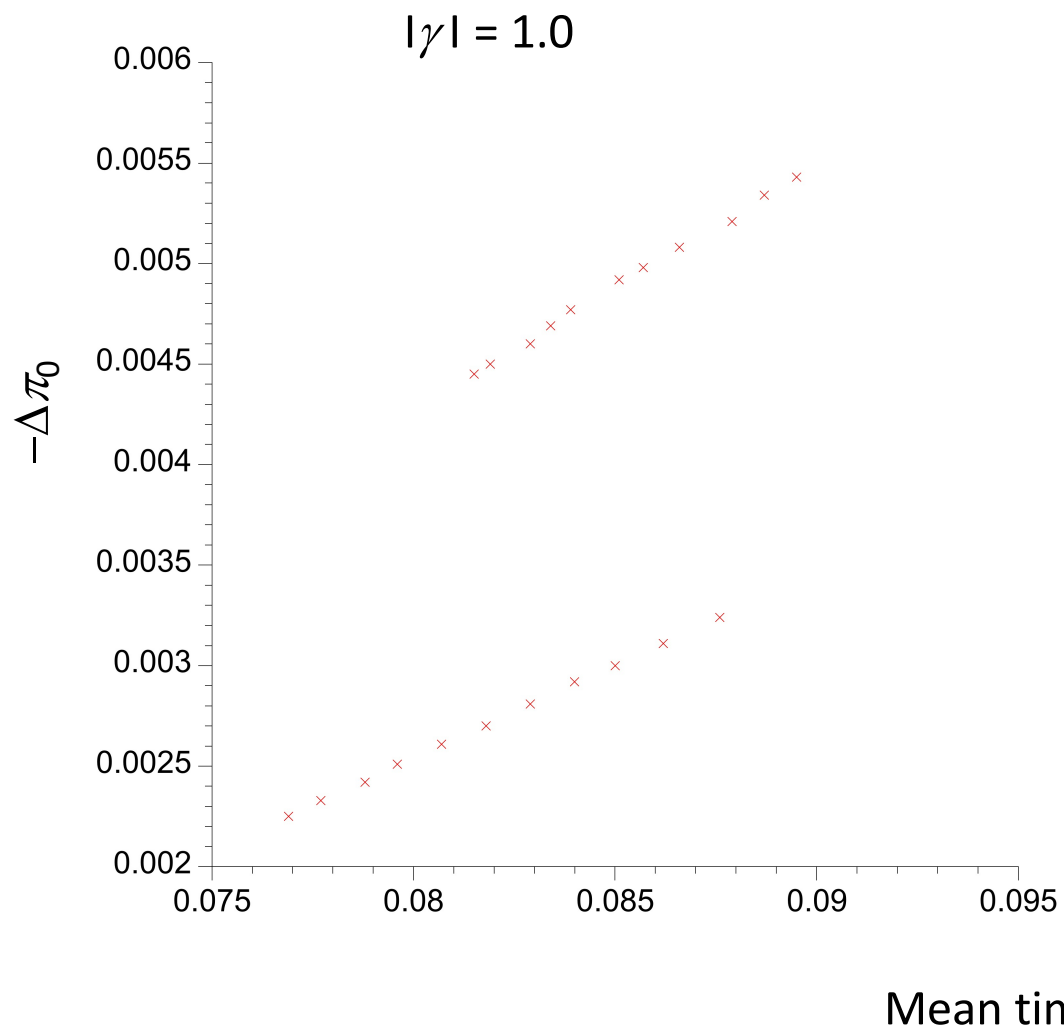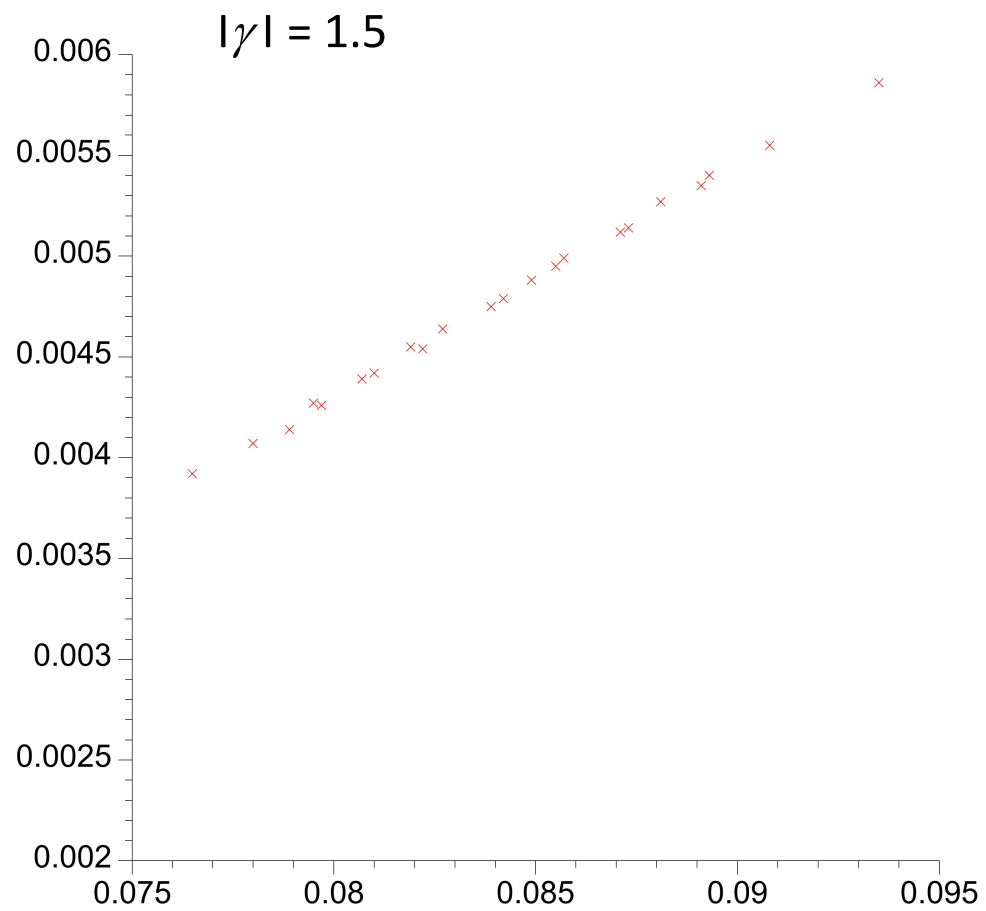

Supplement: iyac027_Supplementary_Data [file iyac027_supplementary_data.zip › Supplemental_Figure_11_GENETICS-2022-305040.pdf]

Mean sum of diversity over course of a loss event

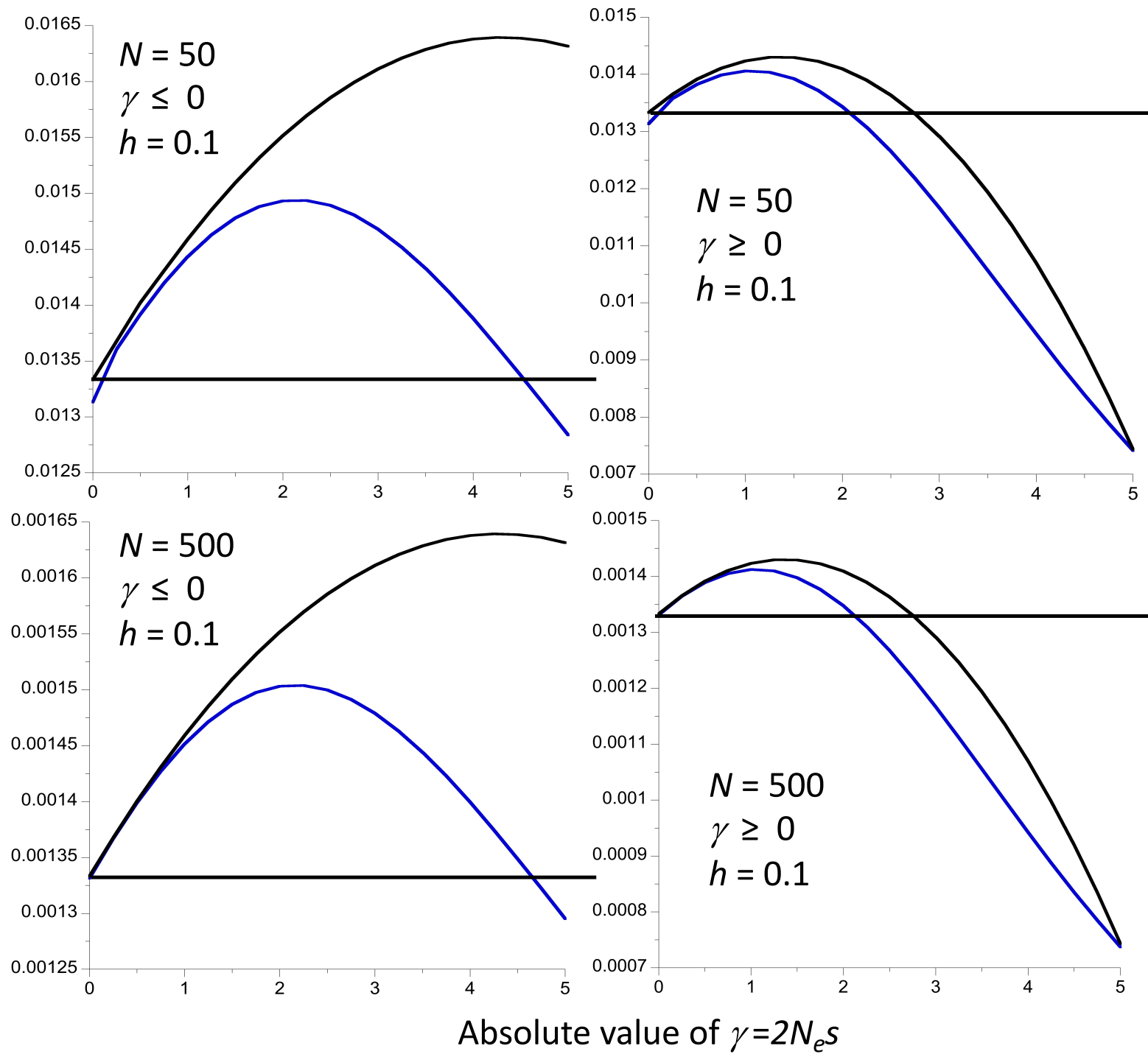

Supplement: iyac027_Supplementary_Data [file iyac027_supplementary_data.zip › Supplemental_Figure_2_GENETICS-2022-305040.pdf]

Mean sum of diversity over course of a fixation event

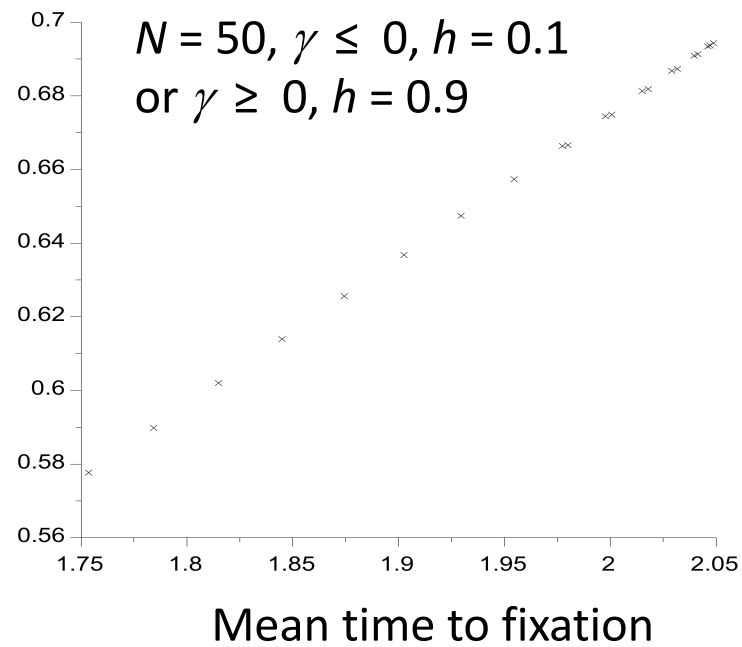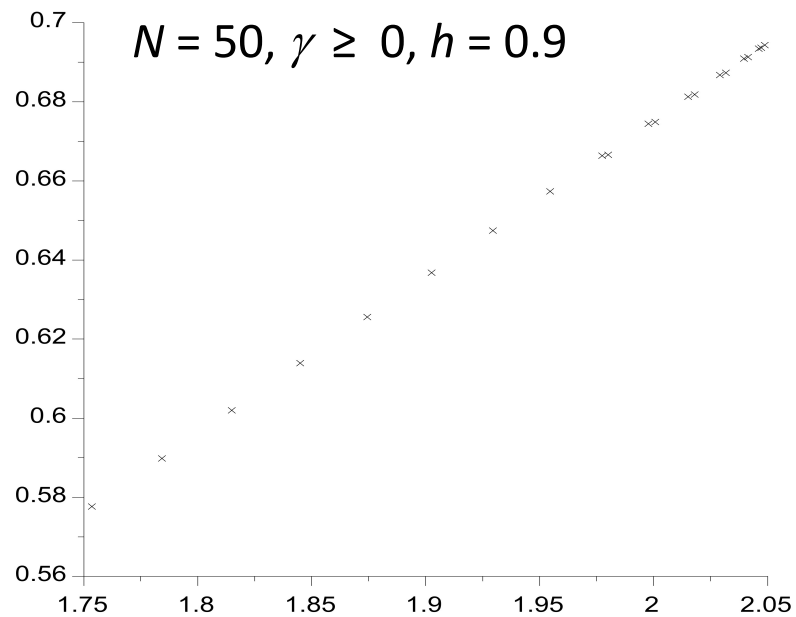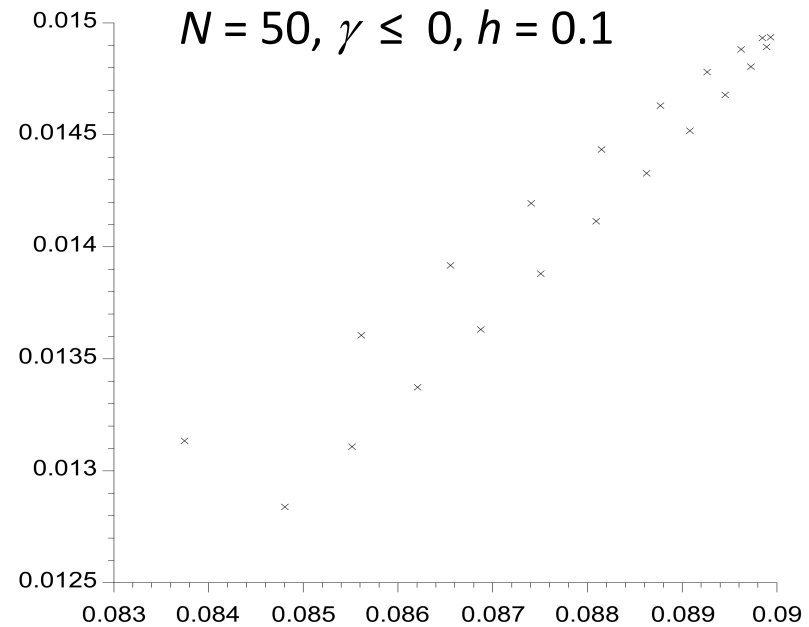

Supplement: iyac027_Supplementary_Data [file iyac027_supplementary_data.zip › Supplemental_Figure_3_GENETICS-2022-305040.pdf]

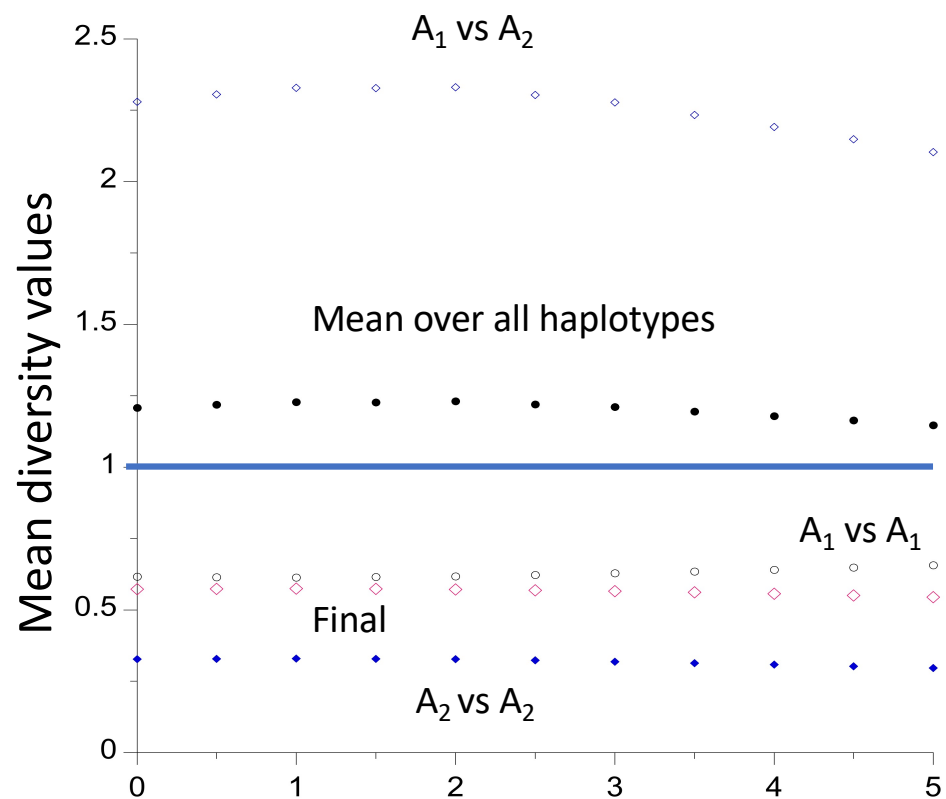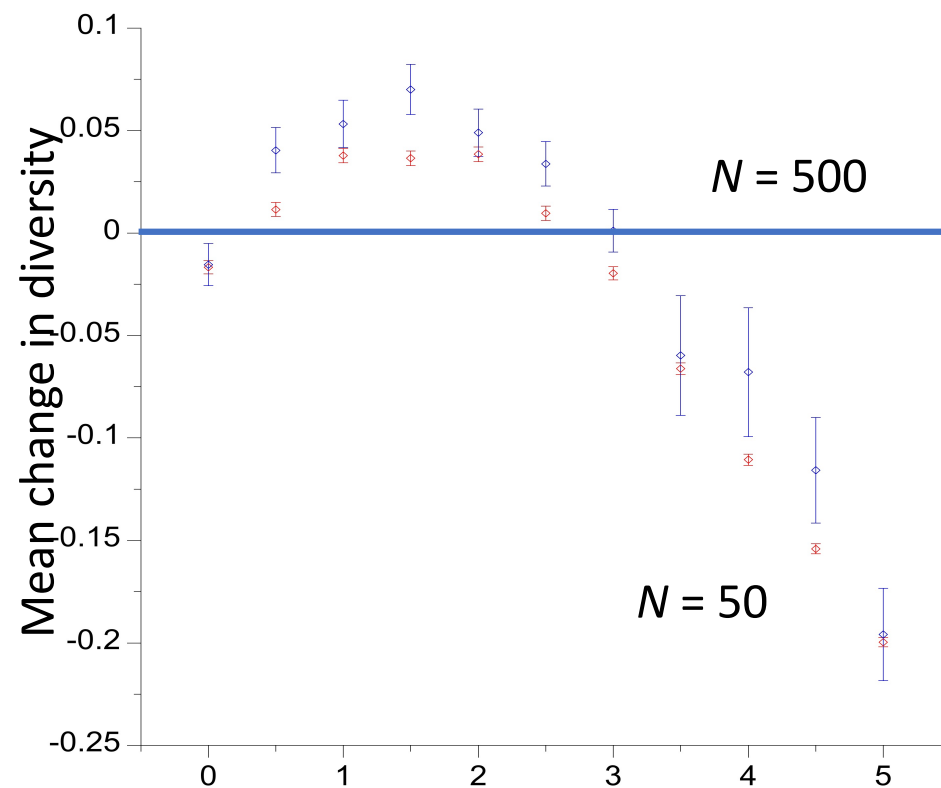

Supplement: iyac027_Supplementary_Data [file iyac027_supplementary_data.zip › Supplemental_Figure_4_GENETICS-2022-305040.pdf]

$$\gamma \geq 0, h = 0.9$$

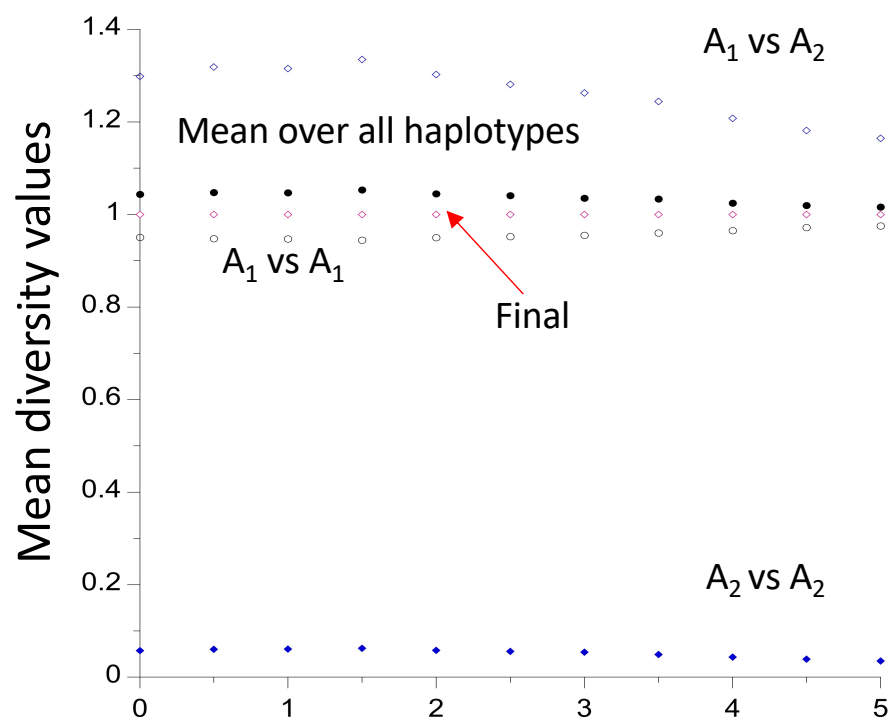

$$\gamma \leq 0, h = 0.1$$

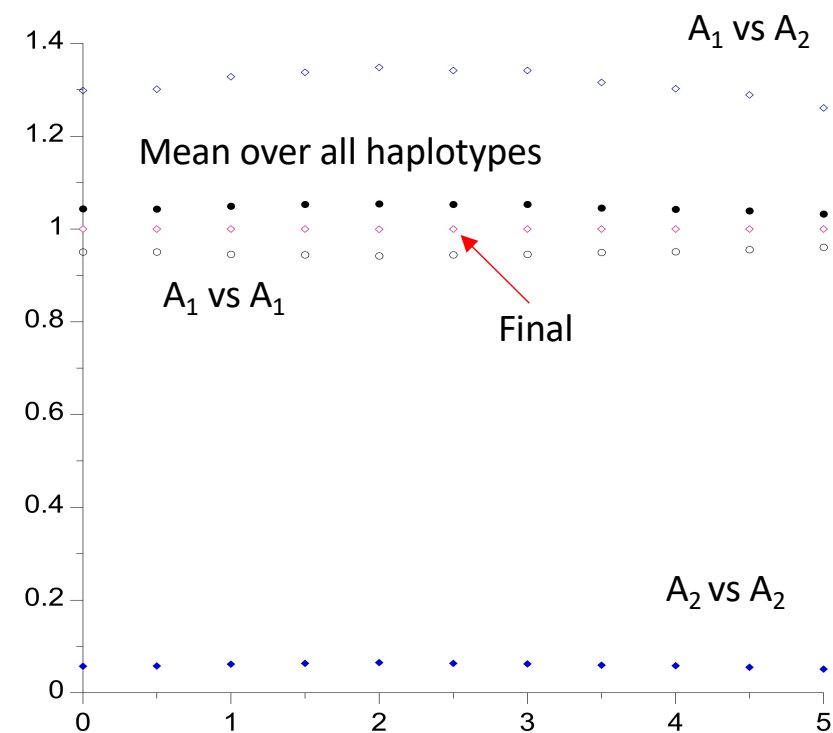

Absolute value of scaled selection coefficient  $\gamma$

Supplement: iyac027_Supplementary_Data [file iyac027_supplementary_data.zip › Supplemental_Figure_5_GENETICS-2022-305040.pdf]

$|\gamma| = 1.0$

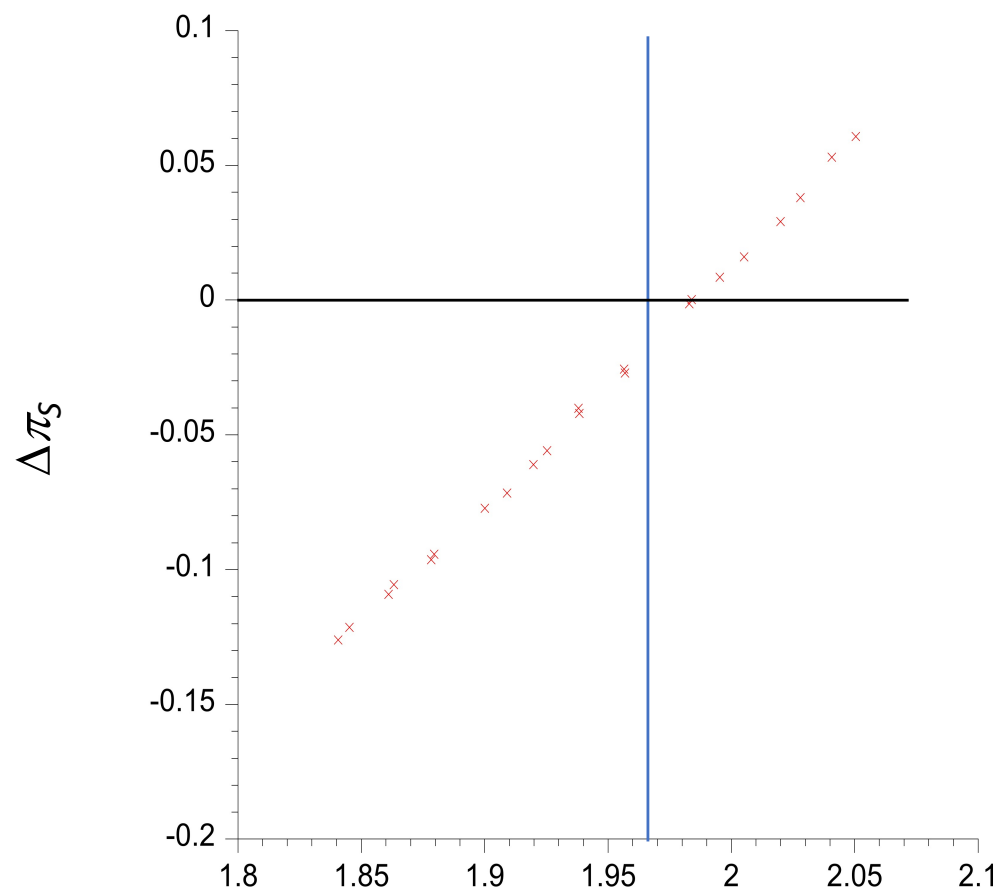

$|\gamma| = 1.5$

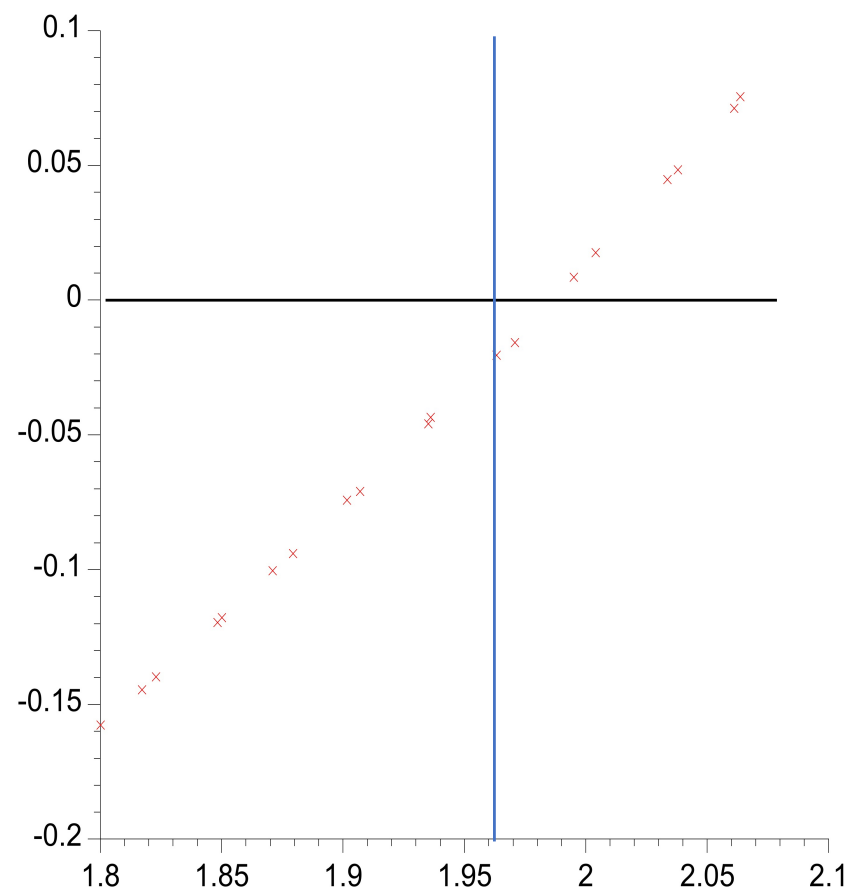

Mean time to fixation

Supplement: iyac027_Supplementary_Data [file iyac027_supplementary_data.zip › Supplemental_Figure_6_GENETICS-2022-305040.pdf]

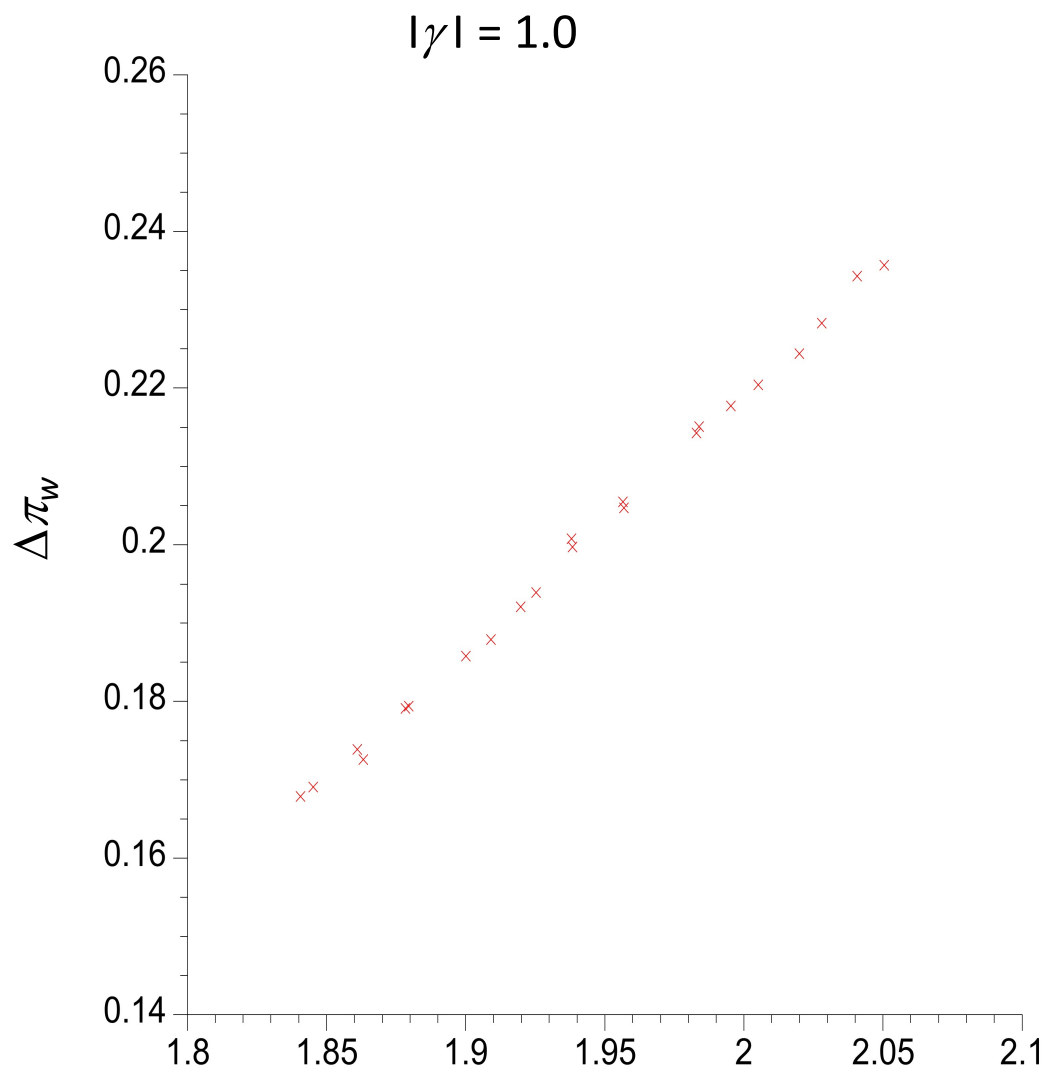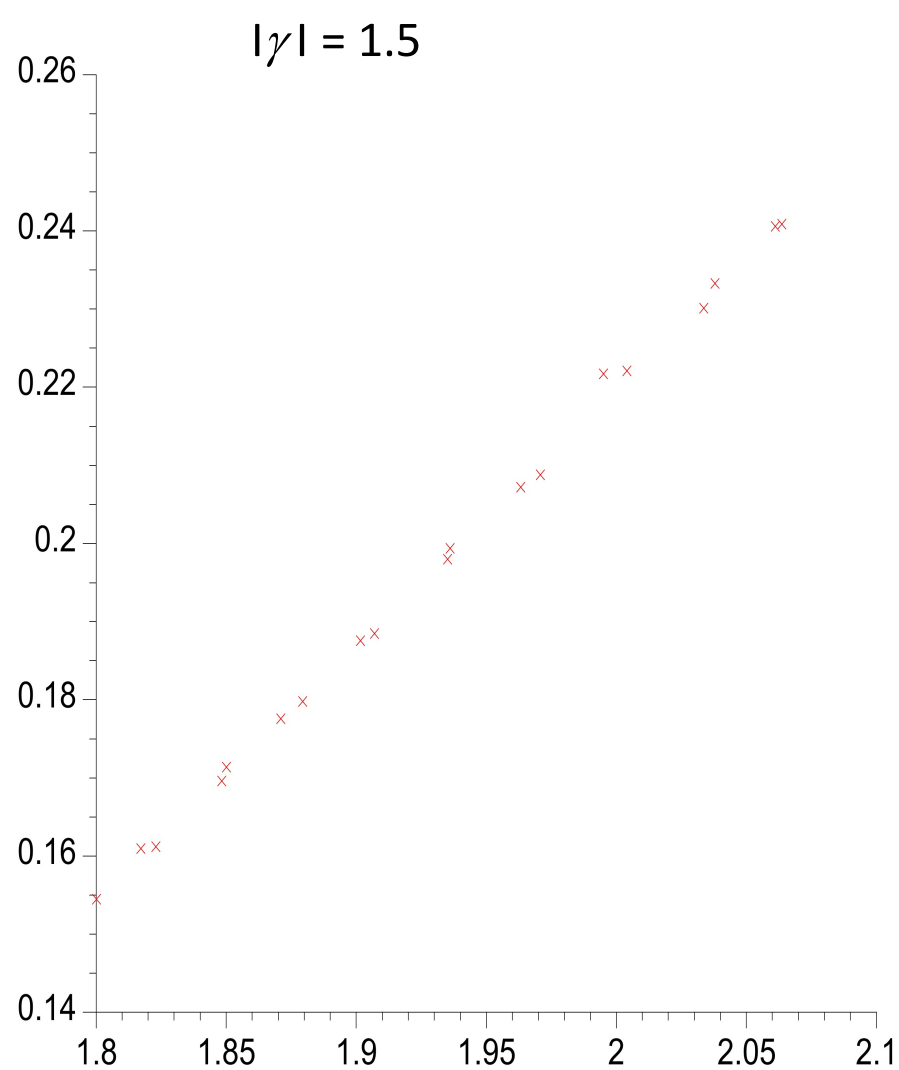

Mean time to fixation

Supplement: iyac027_Supplementary_Data [file iyac027_supplementary_data.zip › Supplemental_Figure_7_GENETICS-2022-305040.pdf]

$|\gamma| = 1.0$

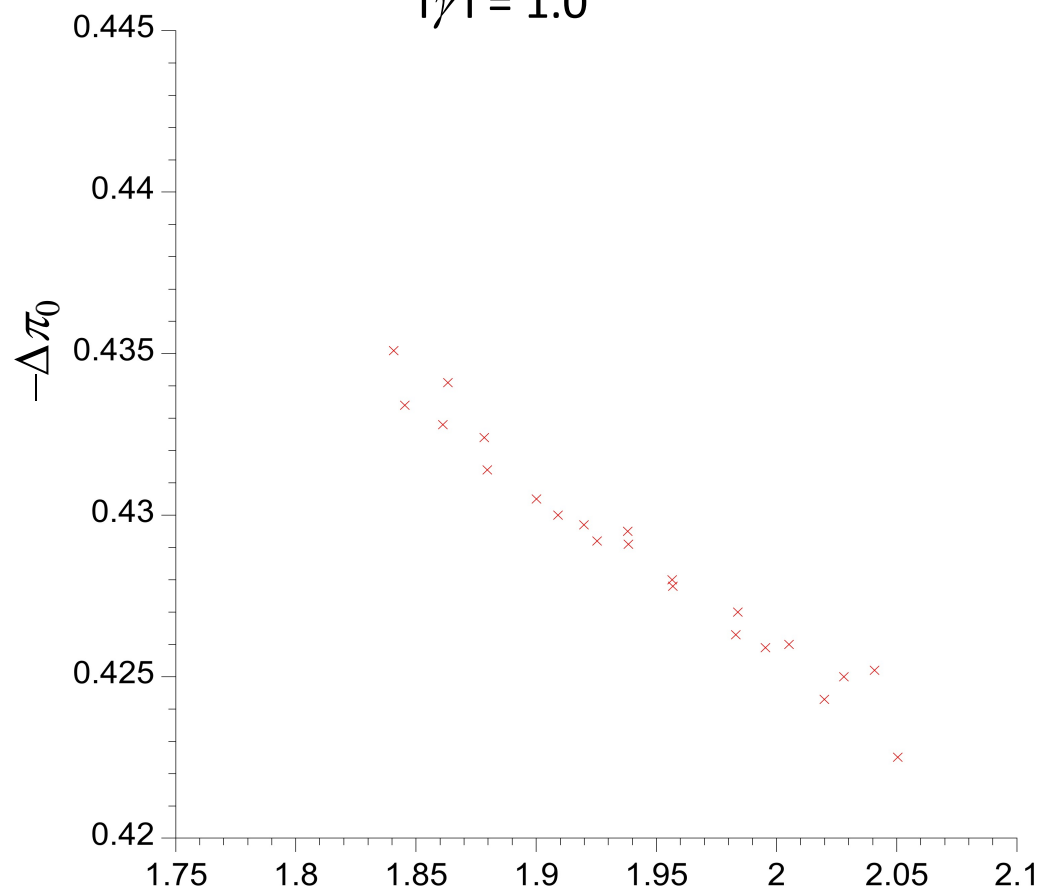

$|\gamma| = 1.5$

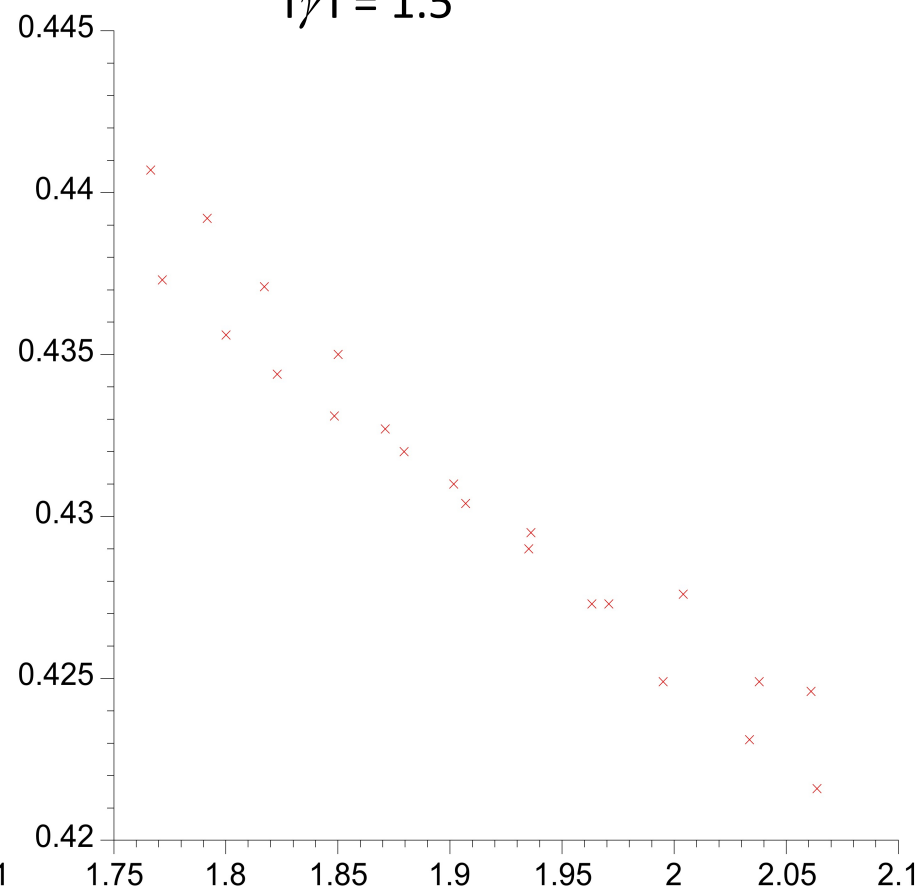

Mean time to fixation

Supplement: iyac027_Supplementary_Data [file iyac027_supplementary_data.zip › Supplemental_Figure_8_GENETICS-2022-305040.pdf]

$|\gamma| = 1.0$

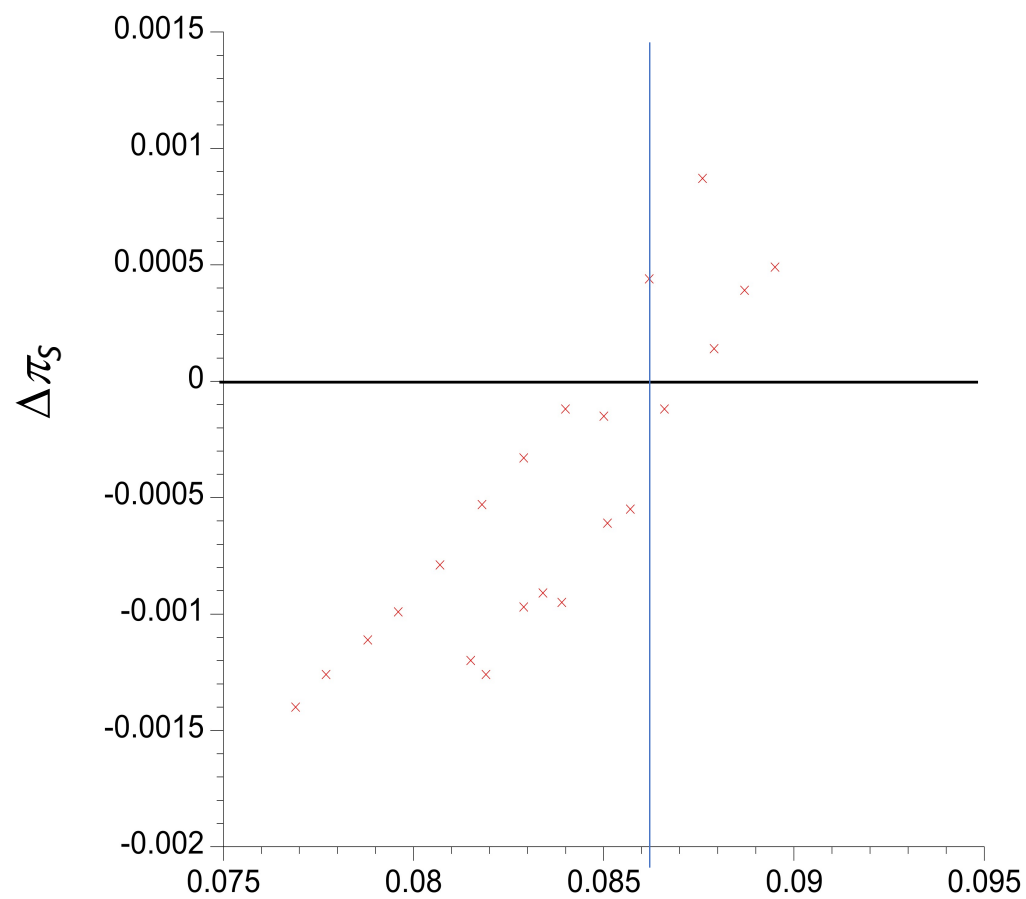

$|\gamma| = 1.5$

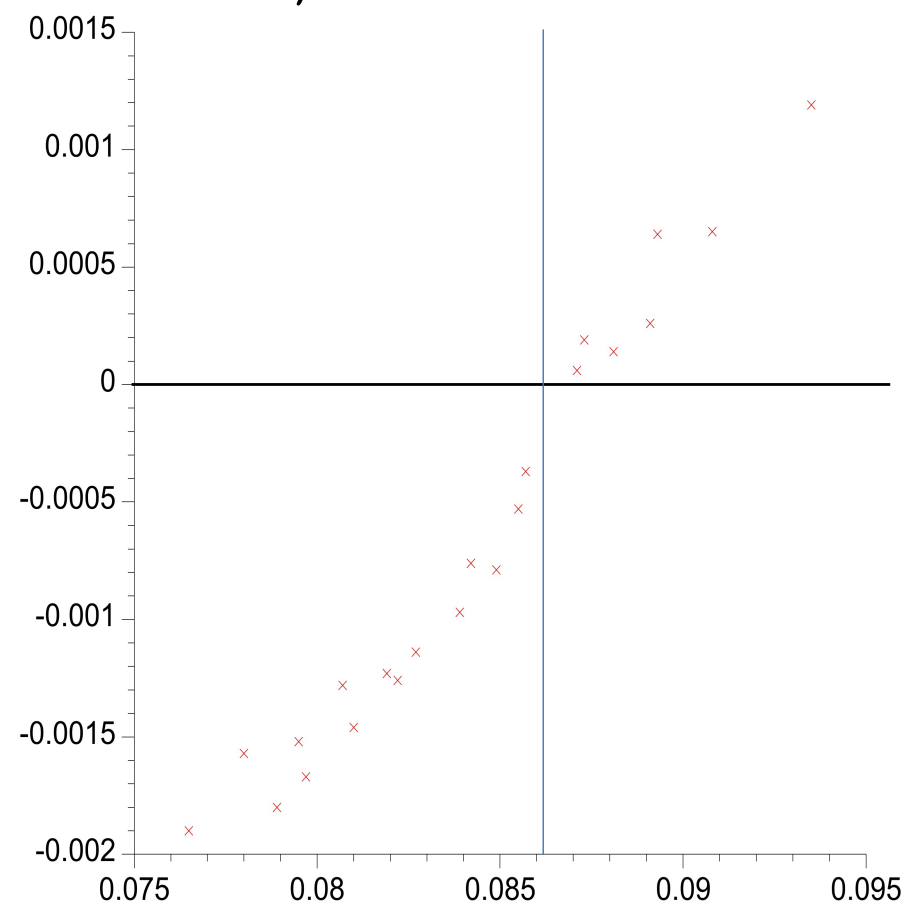

Mean time to loss

Supplement: iyac027_Supplementary_Data [file iyac027_supplementary_data.zip › Supplemental_Figure_9_GENETICS-2022-305040.pdf]
